# Measuring reading and language skill in generation Scotland: Scottish Family Health Study

**DOI:** 10.1136/bmjph-2025-004427

**Published:** 2026-05-21

**Authors:** Hayley Susan Mountford, Archie Campbell, Riccardo Marioni, Timothy C Bates, Heather C Whalley, Michelle Luciano

**Affiliations:** 1 School of Philosophy, Psychology and Language Sciences, The University of Edinburgh College of Humanities and Social Science, Edinburgh, UK; 2 Centre for Genomic and Experimental Medicine, The University of Edinburgh Institute of Genetics and Cancer, Edinburgh, UK; 3 The University of Edinburgh Centre for Clinical Brain Sciences and Centre for Medical Informatics, Edinburgh, Scotland, UK

**Keywords:** Epidemiology, Molecular Epidemiology, Mental Health, Education, Communication

## Abstract

**Introduction:**

Successful acquisition of language and literacy skills is essential to child development and is associated with positive socioeconomic and well-being outcomes later in life. Research into communication skills has primarily focused on early development and childhood. This is particularly the case for studies of genetic variation in reading and language skills, which rarely include older adults; the largest genome-wide association study to date includes participants only up to 26 years of age. We argue that reading-related traits remain stable across the adult lifespan and that including older adults offers a way to increase statistical power for gene discovery. Here, we describe newly available reading, spelling and oral-language-related measures in the Generation Scotland: Scottish Family Health Study (GS:SFHS).

**Methods:**

Phenotypic data in GS:SFHS were extended to include quantitative measures of reading, spelling and language-related measures as well as self-reported neurodevelopmental and psychiatric conditions. Participants also reported frequency of book reading in both childhood and adulthood. Multiple regression analyses were conducted to examine associations between reading-related measures and age and characterise their stability across the adult lifespan.

**Results:**

Reading-related data were collected for N=1595 GS:SFHS participants aged 29.5–76.9 years. Regression analyses indicated that reading and spelling performance were stable across the adult lifespan. In contrast, negative curvilinear effects of age^2^ were observed with phonological verbal-memory, auditory short-term memory and working memory, indicating decreasing performance with increasing age.

**Conclusions:**

These data provide a novel resource for investigating reading, spelling and language skills in adults. The opportunity to link these measures with the existing and future biomarker, cognitive and health record data within GS:SFHS offers a deeply phenotyped dataset with substantial potential for replication studies, meta-analyses and future genetic discovery.

**WHAT IS ALREADY KNOWN ON THIS TOPIC**

- Reading and language skills are key to educational attainment, and while proficiency in these skills is obtained in childhood, we know that difficulties in these domains often persist throughout the lifespan.
- Large-scale adult biobanks rarely contain reading, spelling or language-related measures. Child-based cohorts with available reading and language measures are often far smaller, therefore lacking power for genetic studies.

**WHAT THIS STUDY ADDS**

- This study provides measures of reading, spelling and language in a subset of adult participants from Generation Scotland: Scottish Family Health Study (GS:SFHS) as well as self-reports of neurodevelopmental conditions.

**HOW THIS STUDY MIGHT AFFECT RESEARCH, PRACTICE OR POLICY**

- We show that reading and spelling abilities are stable throughout the lifespan, supporting the use of adult cohorts alongside child cohorts.
- Not including older cohorts is a missed opportunity to increase sample sizes in genetic and epigenetic studies of reading and spelling and to provide the opportunity to link with biomarker, cognitive and health data available in the GS:SFHS cohort to address other important questions.

## Introduction

 Written and spoken language skill varies in the population and a significant part of this variation is due to genetic effects passed down by parents to offspring.^1 2^ Gene discovery for achievement on reading-related tasks lags behind that of other cognitive traits, such as general cognitive function.^3^,^5^ Genetic studies of reading traits tend to focus on children and young adults, whereas cognitive function also includes adult populations in which data collection is easier. Here, we capitalised on existing genetic data in the Generation Scotland: Scottish Family Health Study (GS:SFHS)^6 7^ to collect standardised tests of reading and spelling, measures of phonological verbal-memory, auditory short-term and working memory, self-reported measures of developmental and adult health/well-being, and book reading frequency. With linkage to other data in GS:SFHS, this resource allows researchers to answer important questions about the causes and consequences of communication abilities. GS:SFHS used a family-based design to better separate genetic effects from shared environmental influences by comparing relatives. This reduces confounding and enables more accurate estimation of heritability and causal relationships than studies using unrelated individuals (singletons). While not directly relevant to our current analysis, this design offers valuable potential for future research, such as exploring heritability and within-family effects.^6^,^8^ Additional details on the GS:SFHS study are found in online supplemental notes.

We used the same protocol as used in Doust *et al,*^9^ who targeted adults with available whole genome genotyping data in an Australian cohort. They replicated several previous dyslexia candidate genes in their adult sample for quantitative measures of reading ability, thus supporting adults as a valid demographic in which to study skills typically acquired in childhood. Indeed, maximal reading and writing skills are not attained until young adulthood (mid-20s) although are highly stable from late childhood through young adulthood as well as across middle-aged and older groups.^10^ One caveat is that ageing processes in later life, such as the onset of dementia, can affect verbal skills,^11^ thus we aimed to restrict our sampling in GS:SFHS to adults aged 75 years and younger at the point of initial contact for participation in the reading study. The present study describes the reading and language testing protocol used in GS:SFHS and, importantly, provides descriptive statistics of the primary measures with regards to demographics, their correlations with each other and with external measures of validity. The presence of literacy difficulties in our sample coupled with the richness of linked biomarker, cognitive, personality and health data offers multifaceted research opportunities (beyond the gene discovery research question that motivated this study) to those interested in specific learning difficulties and reading ability more generally.

## Methods

### Generation Scotland: Scottish Family Health Study

GS:SFHS is a large family cohort study of 24 084 individuals from 5501 families across Scotland^8^ (online supplemental notes). Initial GS:SFHS recruitment took place between 2006 and 2010, with participants aged between 35 and 65 years at baseline, with collections of cognitive and health data as well as biological data including genomics, methylomics, proteomics and metabolomics. The median age at baseline was 47 years and the sample was 59% female.^6 8^

### Test battery

GS:SFHS participants who had agreed to be recontacted and were under the age of 75 at the time of recontacting for the reading study were invited to participate in the study (email N=7000, or postal invitation N=2200) beginning November 2020 through to February 2023. Participants were sent an email from Generation Scotland describing the study and containing a link to the recruitment page. They were then presented with the participant study information, gave consent to participate and provided contact details. Participants were asked if they had a hearing impairment that would make it difficult to answer questions over the phone. Individuals who consented to participate were then sent a link to the online self-report questionnaire (online supplemental notes) on the presence of specific learning difficulties, including reading difficulties and psychiatric traits. Participants were asked how frequently they read a book, ranging from less than once a year/never through to every day or about every day. Similarly, participants were asked how frequently they read a book as a child. Participants were asked if they had any biological children, then if yes were they happy to respond to additional questions on those children. If they answered yes, they were asked if any of their biological children had a reading or language difficulty, then provided with free text to report the diagnosis.

Interviewers then contacted participants directly to schedule test administration. If a participant reported a hearing problem that could affect their ability to hear instructions over the phone, they were asked to wear a hearing aid. If this was not possible and the participant was unable to hear adequately, they were excluded at this stage. Participants then completed a telephone interview with a trained interviewer who administered the psychometric reading, spelling and memory tests, lasting between 30 min and 45 min. The choice of telephone data collection was driven by our aim to increase participation and inclusion of participants who are geographically spread throughout Scotland and the wider UK and has been shown to be a valid and reliable means of assessment.^9 12^ Reading ability was assessed using the Castles and Coltheart test 2 adults (CC2A),^13^ spelling ability using the Components of Reading Examination spelling test,^1^ non-word repetition (a marker of phonological short-term memory) using the Children’s Test of Nonword Repetition (CNRep),^14 15^ and auditory short-term and working memory using digit span forwards and letter-number sequencing tests from the Wechsler Adult Intelligence Scale (WAIS)-III.^16^ Additional details on how the tests were conducted by telephone are found in online supplemental notes.

### Data cleaning and quality control

**Figure 1:**
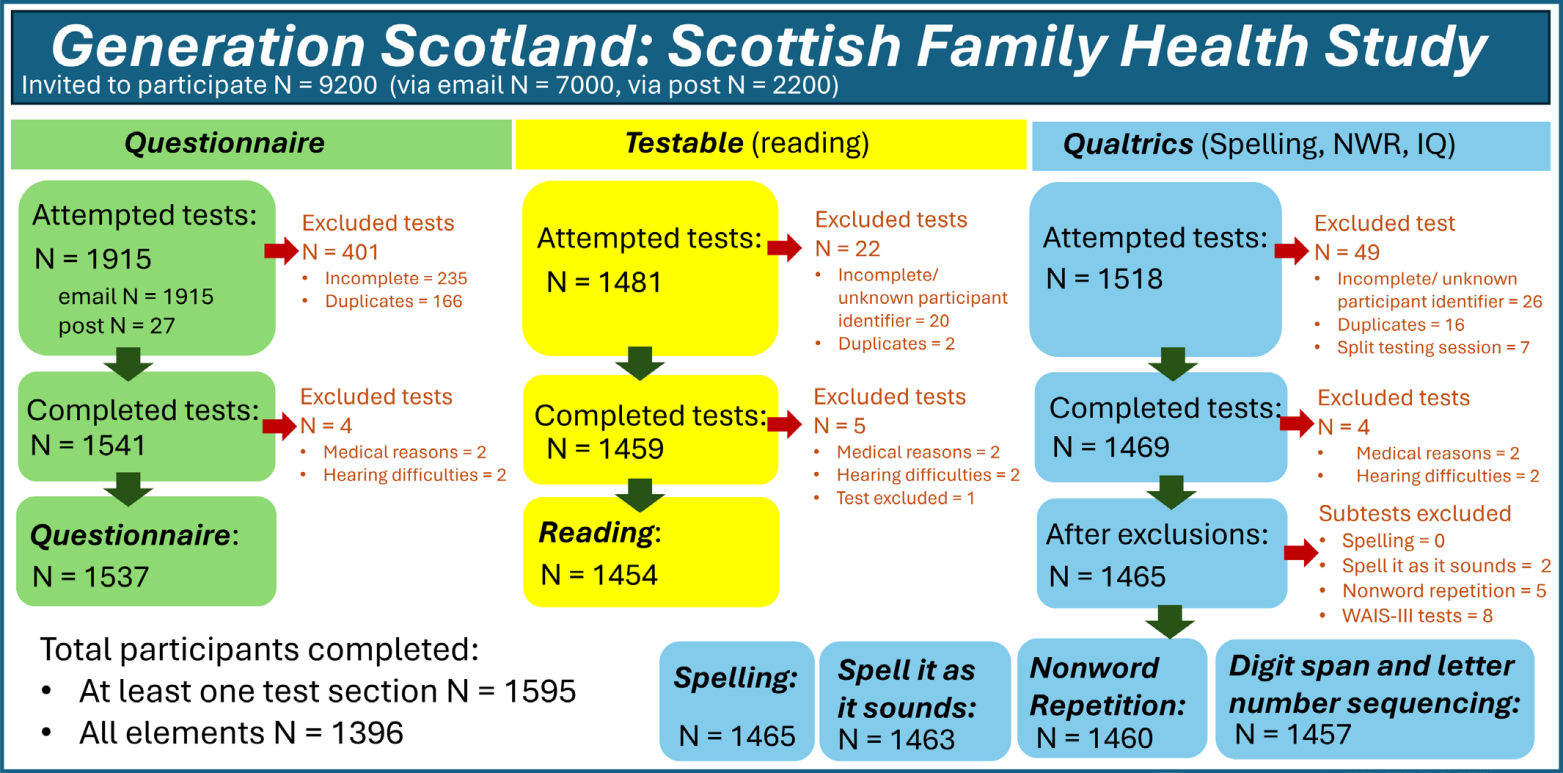
Flowchart of GS:SFHS study participant sampling recruitment and exclusions. GS:SFHS, Generation Scotland: Scottish Family Health Study; IQ, intelligence quota; NWR, non-word repetition.

Test data outputs from Testable (reading), Qualtrics (spelling, spell it as it sounds, non-word repetition, WAIS-III memory subtests) and the self-report questionnaire were cleaned separately using RStudio. Interviewers provided records of testing difficulties, including poor quality telephone lines, repeated tests, internet and software failures that interrupted the testing process. Abandoned and incomplete tests were excluded (figure 1). Descriptive statistics for quantitative measures were generated using R, following exclusions for failed tests but prior to medical conditions detailed below (table 1). Details for all test exclusions and descriptive statistics are presented in figure 1 and online supplemental notes. Extreme outlier scores were retained for reading, spelling, spell it as it sounds and non-word repetition, and winsorised to within ±4 SD of the mean.^17^

**Table 1:** Descriptive statistics for raw quantitative measures

	Completed tests, prior to exclusions		Completed tests following exclusions		
	N	Mean score (SD)	N	Mean score (SD)	Score range (max)
Reading (CC2A) total score	1459	152.38 (10.23)	1454	152.4 (10.1)	73–165 (165)
Reading (CC2A) regular words	–	–	1454	53.4 (2.1)	30–55 (55)
Reading (CC2A) irregular words	–	–	1454	48.9 (3.8)	24–55 (55)
Reading (CC2A) non-words	–	–	1454	50.1 (5.4)	13–55 (55)
Spelling total score	1469	25.32 (3.0)	1465	25.3 (3.0)	4–28 (28)
Spelling regular words	–	–	1465	12.7 (1.5)	2–14 (14)
Spelling irregular words	–	–	1465	12.6 (1.9)	0–14 (14)
Spell it as it sounds	1469	9.8 (2.7)	1463	9.8 (2.7)	0–14 (14)
Non-word repetition	1469	33.9 (5.8)	1460	33.9 (5.7)	7–40 (40)
Digit span forwards (WAIS-III)	1469	11.8 (2.4)	1457	11.8 (2.4)	5–16 (16)
Letter number span (WAIS-III)	1469	10.9 (3.5)	1457	11.0 (3.5)	1–21 (21)

This table shows descriptive statistics for completed raw quantitative measures, before and after exclusions. The number of completed tests (N) and mean scores with SD are shown for before and after exclusions. Score ranges with maximum score indicated in brackets are shown for each test component following exclusions.

CC2A, Castles and Coltheart test 2 adults; WAIS, Wechsler Adult Intelligence Scale.

### Neurodevelopmental and psychiatric conditions binary variables

Reading/writing difficulties: participants were assigned as cases if they self-reported having any difficulties with reading and/or writing (online supplemental notes) for any reason, including eyesight problems, dyspraxia, dyslexia and physical injury or neurological condition impacting handwriting. Participants who had either reading or writing difficulties mentioned in the interviewer notes or free text in the self-report questionnaire were also assigned as reading/writing difficulties cases.

Reading difficulties: participants were assigned as a case if they self-reported reading/writing difficulties as above, and these were also reading specific, that is, not pertaining to writing difficulties (eg, dyspraxia, hand problems).

Dyslexia: more conservatively, participants were assigned as a dyslexia case if they met the criteria for reading difficulties, and either self-reported as having dyslexia or noted by the tester during the interview. If they met the criteria for reading difficulties, but dyslexia was not specified, and they scored >1 SD below the mean on at least two reading or spelling subtests with no evidence of an explanatory condition (eg, writing difficulties, eyesight or other medical conditions), then they were assigned as dyslexia cases.^1 13^

Neurodevelopmental and psychiatric binaries: all other binary neurodevelopmental and psychiatric variables were generated from the self-report, interviewer notes and free text noted elsewhere by the participant. Variables were obtained for stuttering, language impairments, dyspraxia, autism spectrum disorder, attention deficit hyperactivity disorder (ADHD), developmental delay, obsessive compulsive disorder (OCD), depression, social anxiety disorder, eating disorder, personality disorder and bipolar disorder.

### Statistical methods

Multiple regression: quantitative variables were adjusted for sex, age and age-squared (age^2^) using multiple regression (*lm* in R) (*adjusted variable ~sex+age+age^2^*) and visually inspected for normality. Residuals were normalised by inverse normal transformation and rescaled from 0 to 1 (code available at https://github.com/hayley-mountford/GS_reading_study). Plots were generated using *ggplot2*^18^ and *cowplot*^19^ in R.

Phenotype correlations: correlations were generated for continuous variables adjusted for sex, age and age^2^ and normalised, ordinal and binary variables using *mixedCor* (*psych*)^20^ using complete pairs, and visualised with *ggcorrplot*.^21^ False discovery rate (FDR)-corrected p values are reported using the Benjamini-Hochberg method.^22^

Composite measures of reading ability: to reduce multicollinearity among reading-related measures and derive an overall composite score of reading ability, principal component analysis (PCA) was conducted using the *principal* function in the R package, *psych*.^23^ Correlations between measures were r>0.30, indicating sufficient shared variance to justify dimension reduction. PCA was performed without rotation on raw (unscaled but winsorised) residual scores from reading and spelling tests after adjusting for sex, age and age² and restricted to individuals with complete data (N=1466). The number of components to retain was determined by visual inspection of the scree plot using the *psych* package.^23^

Cohort validation: Welch’s two sample t-test (implemented using the *t* test function in R)^24^ was used to compare the means of the normalised quantitative measures between groups defined by self-reported neurodevelopmental and psychiatric conditions. This test was selected as it does not assume equal variance between groups. To account for multiple testing, p values were adjusted using Benjamini-Hochberg method FDR correction as previously described.^22^ The corrected p values are reported. Associations were considered statistically significant at an FDR-adjusted threshold of 0.05. Further details of the statistical tests are provided in online supplemental notes.

Biological children: associations between self-reported reading or language difficulties in study participants and the presence of difficulties in their biological children were evaluated using Fisher’s exact test for count data (implemented by Fisher’s test in R).^25^ This test was selected to account for small sample sizes and low expected counts. P values were reported, and statistical significance was assessed at a threshold of 0.05. Further details of the test, including hypotheses and probability calculation, are provided in online supplemental notes. Association between self-report of reading difficulties or language impairment in at least one biological child and composite reading ability was performed using Welch’s two sample t-test as previously described. Extended statistical methods and interviewer effects are presented in online supplemental notes.

### Patient and public involvement

Patients and the public were not involved in the design, conduct, reporting or analysis of this specific study, although lay members from the University of Edinburgh’s PPI Advisory group gave critical feedback on the participant information and consent sheets. Participants were recruited from the GS:SFHS where they had previously consented to be recontacted for future research participation. Participant consent was given digitally at recruitment and verbally at the beginning of the telephone interview. Patient and public engagement activities including dissemination of research findings are undertaken as part of the wider GS:SFHS research programme.

## Results

Hearing difficulties were reported in 61 participants and 7 were excluded prior to commencing the study as their ability to hear instructions over the phone was impacted. Participants were instructed to wear their hearing aid if required. Interviewers noted two participants were unable to hear sufficiently, and two reported medical conditions impacting their ability to be tested and were therefore excluded post-testing (N=4).

1595 participants (67.5% female) completed at least one of the test sections. Age ranged 29.5–76.9 years (mean=60.3, SD=11.0), and participants were 75 year or younger at initial recruitment to the reading study. Socioeconomic status was measured by the Scottish Index of Multiple Deprivation (SIMD) rank obtained at baseline, which ranges from the most deprived (rank 1) to the least deprived (rank 6505). The mean SIMD rank for this cohort was 4259 (SD=1711), slightly lower than the wider GS:SFHS cohort (mean=4331, SD=1958); 20% of the participants came from the most deprived 30% of SIMD ranks (between 1 and 1952).^26^ Of the 1595 participants, 420 people were related to at least one other person, and 195 family clusters were present. Two-hundred and seventy-eight participants had a first-degree relative (parent/child, sibling or half-sibling) also take part.

1454 participants (67.5% female) completed the CC2A reading test (aged 29.4–76.9 years, mean=60.8, SD=10.8), 1465 individuals (67.4% female) completed the Qualtrics test section (spelling, spell it as it sounds, non-word repetition and intelligence quota (IQ); aged 29.4–76.9 years, mean=60.8, SD=10.8) and 1537 participants (67.53% female) completed the online self-report questionnaire (aged 28.4–76.7 years, mean=60.1, SD=11.1) (figure 1 and online supplemental table 1). All three test components were completed by 1396 participants (67.41% female), ranging 29.5–76.9 years (mean=60.7, SD=10.8) (online supplemental table 1).

Tester notes recorded during the interviews identified three additional participants who self-reported dyslexia (and therefore also reading difficulties and reading and/or writing difficulties) and one participant reported a language impairment. This brought the total to 1540 participants: 144 self-reported reading and/or writing difficulties, 92 reported reading difficulties only (ie, specific to reading not writing) and 32 reported dyslexia. Of the 1538 participants who responded to the question on language impairments, 46 indicated they had a language impairment. The descriptive statistics for the quantitative measures are shown in table 1 and the discrete variables in table 2.

**Table 2:** Percentage frequencies of cases for discrete variables

	N	N cases	Percentage cases
Self-reported reading and/or writing difficulties	1540	144	9.35
Self-reported reading and/or writing difficulties diagnosed by:	144	–	–
Clinically diagnosed		24	16.7
Noticed by others (eg, teachers, parents)		48	33.3
Self-evaluated		65	45.1
Unknown		7	4.9
Reading difficulties	1540	92	6.0
Dyslexia	1540	32	2.0
Other language impairment	1538	46	3.0
Other language impairment diagnosed by:	46	–	–
Clinically diagnosed		7	15.2
Noticed by others (eg, teachers, parents)		28	60.9
Self-evaluated		7	15.2
Unknown		4	8.7
Stutter/stammer	1537	41	2.7
Dyspraxia (developmental coordination disorder)	1537	5	0.3
Autism spectrum disorder	1537	3	0.2
Attention deficit hyperactivity disorder (ADHD)	1537	4	0.3
Developmental delay	1537	3	0.2
Obsessive compulsive disorder (OCD)	1537	8	0.5
Depression	1537	334	21.7
Social anxiety disorder	1537	42	2.7
Eating disorder	1537	21	1.4
Bipolar disorder	1537	6	0.4
Personality disorder	1537	5	0.3
Frequency of book reading now:	1537		
Every day or about every day		606	39.4
Several times a week		226	14.7
Several times a month		167	10.9
Several times a year		373	24.3
Less than once a year/never		165	10.7
Frequency of book reading as a child:		1537	
Every day or about every day		453	29.5
Several times a week		322	20.9
Several times a month		285	18.5
Several times a year		305	19.9
Less than once a year/never		172	11.2
Participants with biological children:		1537	
Yes		1222	79.5
No		313	20.4
Did not answer		2	0.1
Child diagnosed with reading impairment:		1222	
Yes		171	14.0
No		1049	85.8
Did not answer		2	0.2
Child diagnosed with a language impairment:		1222	
Yes		54	4.4
No		1166	95.4
Did not answer		2	0.2

Distributions of the raw quantitative measures are presented in online supplemental figures 1–6. Extreme outliers for regular word reading (N=13), irregular word reading (N=8), non-word reading (N=9), regular spelling (N=6), irregular spelling (N=14), spell it as it sounds (N=0) and non-word repetition (N=2) were winsorised to within ±4 SD of the mean.

### Age and sex effects

Multiple regression was used to test the effects of sex, age and age^2^ (online supplemental table 2). Females scored higher on regular (β=0.22, p=0.005) and irregular word spelling (β=0.31, p≤0.001). A negative curvilinear effect of age^2^ was observed for non-word repetition occurring at age 39.42 years (p=0.002), digit span at 53.00 years (p=0.02) and letter-number sequencing at 45.61 years (p=0.02), showing a decrease in scores with ageing (online supplemental table 2). Distributions of quantitative variable scores against age are presented in online supplemental figure 7. To achieve normality, residuals were inverse-normal transformed, rescaled to between 0 and 1 for analysis.

### Phenotype correlations

**Figure 2:**
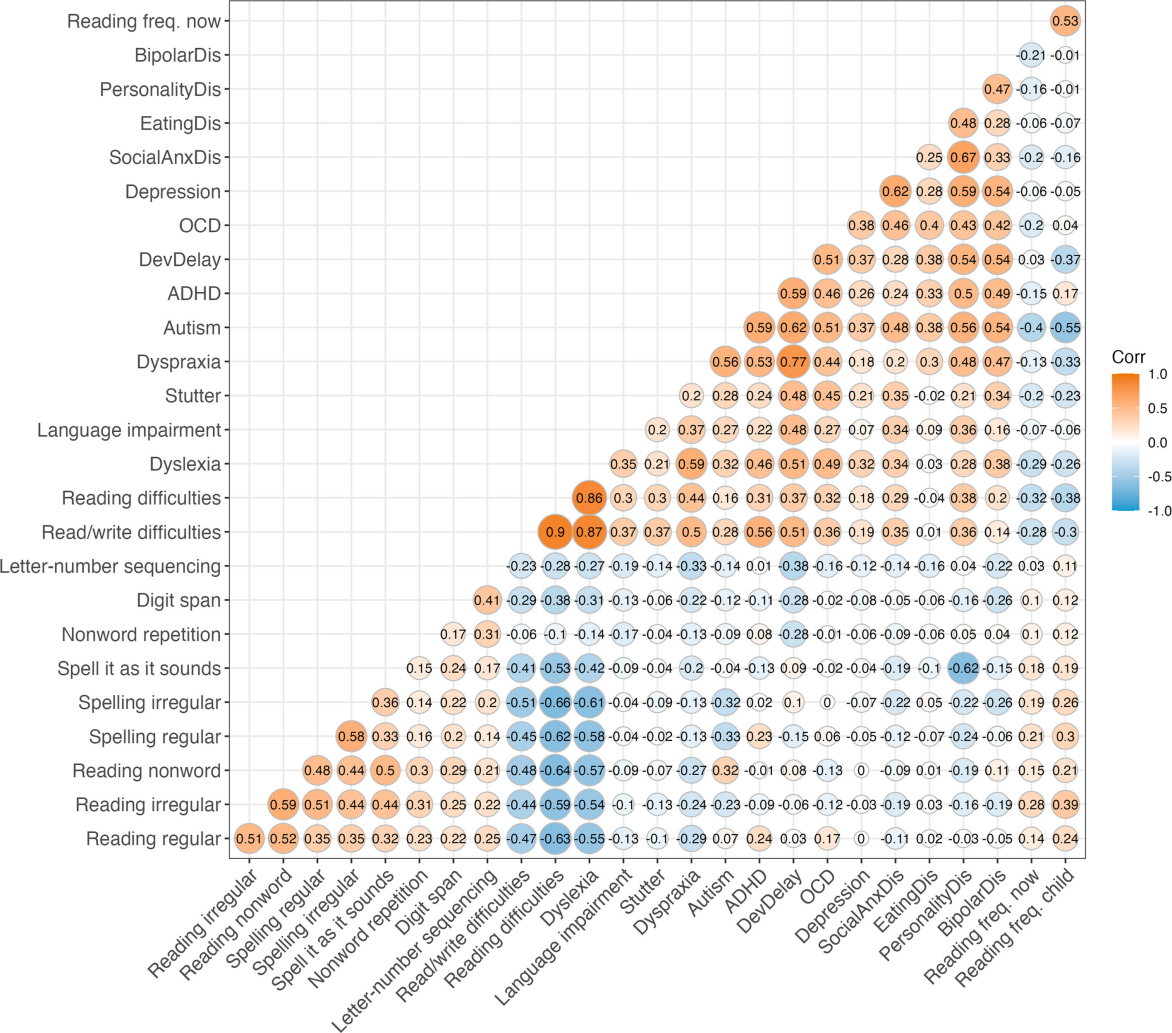
Intercorrelations (Pearson’s r) between adjusted quantitative and discrete measures (N=1454–1595). Correlations are not adjusted for family relatedness. ADHD, attention deficit hyperactivity disorder; OCD, obsessive compulsive disorder.

Figure 2 (online supplemental tables 3 and 4) shows the correlations between unadjusted measures of reading, spelling, phonological verbal-memory and working memory with self-reported diagnoses and frequency of book reading (N=1454–1594). Higher scores in reading measures were moderately correlated with higher skill in spelling (0.35–0.51), after FDR correction. Spell it as it sounds was correlated with higher scores in reading and spelling (≥0.32) and non-word reading (0.5).

Self-reported reading and/or writing difficulties (N=144), reading difficulties (N=92) and dyslexia (N=32) were significantly correlated with poorer performance on reading and spelling (≤−0.44, p≤7.6×10^−5^); and negatively correlated with spell it as it sounds (≤−0.41, p≤5.09×10^−9^), digit span (≤−0.29, p≤1.62×10^−3^) and letter-number sequencing (≤−0.23, p≤1.14×10^−3^) but were not significantly correlated with non-word repetition.

Self-reports of language impairments (N=46) and stuttering (N=41) were not significantly correlated with quantitative measures of reading or spelling. Language impairments showed a significant correlation with lower scores on non-word repetition (−0.17, p=0.02), considered a marker of phonological short-term memory, and with a lower score on letter–number sequencing (−0.19, p=2.06×10^−3^), a marker of verbal working memory, whereas stuttering did not. Self-reports of language impairments were significantly correlated with reading and/or writing difficulties (0.37, p=5.24×10^−4^), reading difficulties (0.3, p=0.02) and dyslexia (0.35, p=0.04), while stuttering was correlated with reading and/or writing difficulties (0.37, p=6.256×10^−4^) and reading difficulties (0.3, p=8.6×10^−3^), but not dyslexia. Five or fewer cases of dyspraxia (N=5), autism (N=3), ADHD (N=4) and developmental delay (N=3) were insufficient for reliable analysis.

Depression (N=334) (−0.12, p=2.06×10^−3^) and eating disorders (N=21) (−0.16, p=0.03) related to lower scores on letter–number sequencing. Social anxiety disorder (N=42) related to lower scores on irregular word reading (−0.19, p=0.03), irregular spelling (−0.22, p=2.41×10^−3^) and spell it as it sounds (−0.19, p=0.02). Self-reports of reading and/or writing difficulties, reading difficulties, dyslexia and stuttering showed moderate correlations with depression (≥0.18, p≤0.03) and social anxiety disorder (≥0.29, p≤0.04). Language impairments were correlated with social anxiety disorders (0.34, p=0.04) but not depression. Correlations between the frequency of book reading and other variables are presented in online supplemental notes.

### Reading ability composite

As reading and spelling measures were highly correlated, we performed a PCA to derive a composite measure of reading ability from regular, irregular and non-word reading, regular and irregular spelling measures adjusted for sex, age and age^2^ (N=1466). The first unrotated component explained 66.6% of variance in the data (online supplemental table 5) (scree plot in online supplemental figure 8), it was extracted, inverse normal transformed and scaled from 0 to 1 for further analysis.

### Cohort validation

Participants with reading difficulties consistently performed significantly lower (FDR-adjusted p≤3.3×10^−14^) than controls on the regular word reading (d=1.39), irregular word reading (d=1.29), non-word reading (d=1.41), regular spelling (d=1.37) and irregular spelling (d=1.48). Participants with either self-reported reading and/or writing difficulties or a diagnosis of dyslexia showed a similar pattern of significantly lower reading and spelling scores (d≥0.91, p≤2.69×10^−5^) (online supplemental table 6). The three groups of individuals with reading difficulties scored consistently lower on Spell it as it sounds (d≥0.84, p≤3.07×10^−7^), digit span (d≥0.51, p≤6.3×10^−3^) and letter–number sequencing (d≥0.46, p≤5.09×10^−3^), but not for non-word repetition (d≥0.12, p≥0.30).

Individuals with dyspraxia (d=1.01, p=0.02) or depression (d=0.21, p=9.49×10^−4^) performed significantly worse on letter–number sequencing. Participants self-reporting social anxiety showed significantly lower scores on spelling irregular words (d=0.53, p=6.15×10^−3^) and Spell it as it sounds (d=0.44, p=0.05). The groups of participants who self-reported stuttering, ADHD, autism, developmental delay, OCD, eating disorder, personality disorder or bipolar disorder did not perform significantly differently to the control groups on any of the psychometric tests, after FDR correction.

### Reading and language impairments in the biological children of participants

Of the 1537 participants who answered the online questionnaire, 79.7% said they had at least one biological child (N=1222). Of the group with biological children, 14% (N=171) said that at least one of their biological children had been diagnosed as having reading impairment and 4.4% (N=54) with language impairment (table 2). Participants who self-reported as having reading and/or writing difficulties were more than three times more likely to report having at least one child with a reading impairment (p=1.23×10^−6^, OR=3.31, 95% CI (2.03 to 5.34)) (online supplemental table 7). Participants who self-reported as having reading difficulties were more than 3.5 times more likely to have at least one biological child with a reading impairment (p=1.19×10^−5^, OR=3.54, 95% CI (1.98 to 6.2)), and participants with dyslexia were more than 2.5 times more likely (p=0.03, OR=2.57, 95% CI (1.67 to 7.73)). If the study participant reported that they had a language impairment, they were 3.6 times more likely to have a child with a reading impairment (p=6.32×10^−4^, OR=3.66, 95% CI (1.67 to 7.73)). They were also 2.8 times more likely to have a biological child with a language impairment, although this did not meet the threshold for significance (p=0.07, OR=2.83, 95% CI (0.7 to 8.44)).

Conversely, we wanted to know if the cohort members who had a biological child with reading difficulties or language impairment scored lower on the overall composite measure of reading ability. We found that parents of children with a reading impairment scored statistically poorer on the overall composite measure of reading ability (d=0.29, p=1.42×10^−03^), whereas parents of children with a language impairment did not (d=0.11, p=0.51) (online supplemental table 8).

Interviewer effects are reported in online supplemental notes, online supplemental figure 9 and online supplemental tables 9 and 10.

## Discussion

We collected detailed reading, spelling and language-related measures along with self-reports of neurodevelopmental and psychiatric conditions in a subset of GS:SFHS participants. In this sample, there was a higher frequency of females (67.5%) than in the full GS:SFHS cohort (59%), and 20% of participants came from the most deprived 30% of SIMD areas versus 17% in the full GS:SFHS cohort.^26^ There was substantial variation in the quantitative measures of reading and spelling, and we showed that attainment on these measures was not influenced by age, and that they were valid markers of reading difficulties/dyslexia, which onset in childhood. As expected, there was overlap with other self-reported developmental traits/disorders and book reading frequency, which further established their validity.

Women scored significantly higher than men on both regular and irregular word spelling, which is consistent with previous findings of a sex difference in reading ability.^27^ We confirmed that there was no effect of age on measures of reading or spelling, which we argued should have reached their maximal attainment by young adulthood. Additionally, because our sample included older participants, it was important to test for the presence of ageing effects on cognitive abilities—which we could rule out. The absence of age effects in this sample aligns with those from an Australian adult cohort^9^ and further supports the suitability of adult cohorts for use in gene discovery of neurodevelopmental traits. Non-word repetition and digit span, both measures of verbal short-term memory, and letter–number sequencing, a measure of verbal working memory, showed a decline in scores for adult participants (non-word repetition at 39.42 years, digit span at 53 years, letter–number sequencing at 45.61-year old). This is consistent with the prior literature,^10 11^ and therefore these measures are less suited for combined analysis with cohorts of children.

We found an underrepresentation of participants self-reporting neurodevelopmental conditions. The expected occurrence rate of dyslexia is around 7%,^28 29^ however estimates vary widely depending on individual cut-offs in continuous measures, comparison to peers and variability in diagnostic procedures.^30^ Only 2% of the cohort reported dyslexia and 6% reported having difficulties with reading. This is most likely due to underdiagnosis of dyslexia in the adult Scottish population, coupled with the difficulties in recruiting participants with reading difficulties to a reading and language study. Notably, the prevalence of diagnosed reading difficulties in children of participants was 14%, and 4.4% for language impairment, confirming generational differences in awareness and screening/diagnosis, especially of dyslexia. The self-reported reading difficulties variable showed stronger prediction of having a biological child with reading impairment than did the dyslexia diagnosis self-report fits this interpretation. Relatedly, parents of children with reading impairment had lower general literacy scores. Quantitative measures of reading and spelling, and the reading difficulties binary variable were significantly correlated with dyslexia and can be used as proxy measures of dyslexia to increase statistical power. Self-reports of dyslexia, reading difficulties and reading/writing difficulties showed strong correlations with self-reports of other neurodevelopmental conditions and are consistent with the emerging literature on co-occurrence and shared genetic aetiology.^2 31 32^ As further validity, frequency of book reading in adulthood (≥0.14) and childhood (≥0.2) were moderately correlated with higher scores in reading and spelling, and with fewer reading difficulties (≤−0.26), which is expected from past literature.^33^

Non-word repetition, a measure of phonological working memory and processing and a proxy for language variation, was affected by age, showing decline in scores after 39 years. It was also the task with the greatest test measurement error as indicated by the presence of interviewer effects. These age and testing bias effects likely explain why non-word repetition was not more highly correlated with dyslexia diagnosis (−0.14 not significant) or language impairment (−0.17, FDR p=0.02). It is well known that reading and language abilities overlap^28^ and that non-word repetition is a sensitive marker of developmental language disorder (DLD) in children.^34 35^ In the Brisbane Adults Reading Study, correlations between non-word repetition and self-reported reading (0.00) and language difficulties (−0.02) were even lower than the present study.^9^ It may be that in adults, this is not an optimal index of past or present language difficulties, but there has been no need to study this in adults because the signs of DLD are typically noticeable in the early stages of language development unlike dyslexia where it more often goes unidentified in adults.

Several methodological limitations should be acknowledged for this study. Conducting the interviews by telephone meant that testing conditions could not be fully controlled, and therefore the data may be noisier than in-person data collection. Technical issues and poor telephone line quality led to the exclusion of several tests, yielding a higher failure rate than in-person testing. Severity of hearing difficulties was not included as a specific variable in the online questionnaire or test battery, and so the impact of these could not be investigated. Prior to commencing the study, participants reported the hearing difficulties in their initial contact with the study (N=61), and interviewers confirmed participants could hear (ie, wearing hearing device, quiet environment, using their other ear). Participants who could not hear sufficiently (N=7) were excluded prior to testing. In the telephone testing session interviewers confirmed participants could hear the instructions, and difficulties were noted. Post-testing, two participants were excluded due to hearing difficulties (N=2) and a further two were excluded due to medical conditions that affected test performance (N=2). Language ability was assessed only through phonological working memory and processing (non-word repetition) and auditory short-term and working memory (digit and letter–number span; WAIS-III). As these measures do not assess syntactic, morphological, semantic or pragmatic skills, they serve only as proxies for oral language competency. It is important to note that we cannot distinguish between individuals who report reading difficulties due to dyslexia and those whose difficulties may reflect inadequate classroom instruction or lack of intervention in childhood. Given that 18% of adults in the UK are considered functionally illiterate and struggle to understand more than short texts and simple contextual information,^36^ barriers to literacy in the population remain substantial. Family relatedness was not modelled in the regression and PCA analyses but given that results were in line with past findings, this is unlikely to have produced significant bias.

## Conclusion

In summary, these data provide a detailed characterisation of reading, spelling and related cognitive measures in a well-phenotyped adult cohort. The stability of reading and spelling performance across adulthood supports the use of adult samples for investigating the genetic basis of reading-related traits. The availability of both quantitative literacy measures and self-reported neurodevelopmental traits further enables the use of proxy phenotypes to increase statistical power in genetic studies. When combined with the extensive biomarker, cognitive and health record data available in GS:SFHS, this resource offers a valuable opportunity for future genome-wide and epigenome-wide association studies and for research in socioeconomics and health.

## Supplementary material

10.1136/bmjph-2025-004427 online supplemental file 1

10.1136/bmjph-2025-004427 online supplemental file 2

10.1136/bmjph-2025-004427 online supplemental file 3

## Data Availability

Data are available upon reasonable request.
