# Supplementary material for: Measuring reading and language skill in generation Scotland: Scottish Family Health Study
Source: BMJ Public Health. 2026 May 21;4(2):e004427. doi: 10.1136/bmjph-2025-004427 (PMC13202090; doi:10.1136/bmjph-2025-004427)
Supplement: online supplemental file 1 [file bmjph-4-2-s001.docx]

**SUPPLEMENTARY FIGURES**


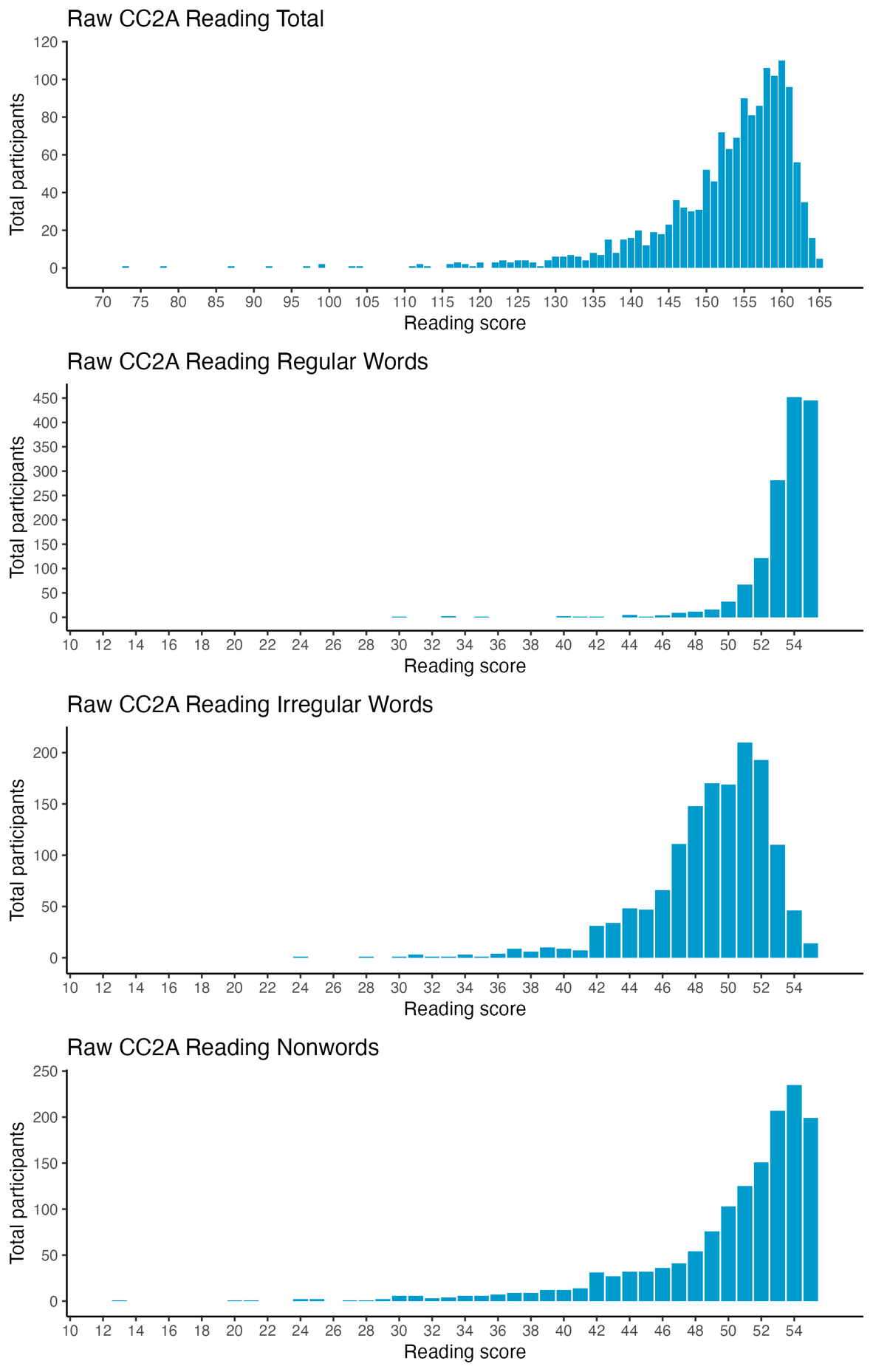
 **Supplementary Figure 1** Distributions of raw CC2A reading test scores, prior to outlier winsorisation. a) Total CC2A score, b) regular words score, c) irregular words score and d) nonwords score.


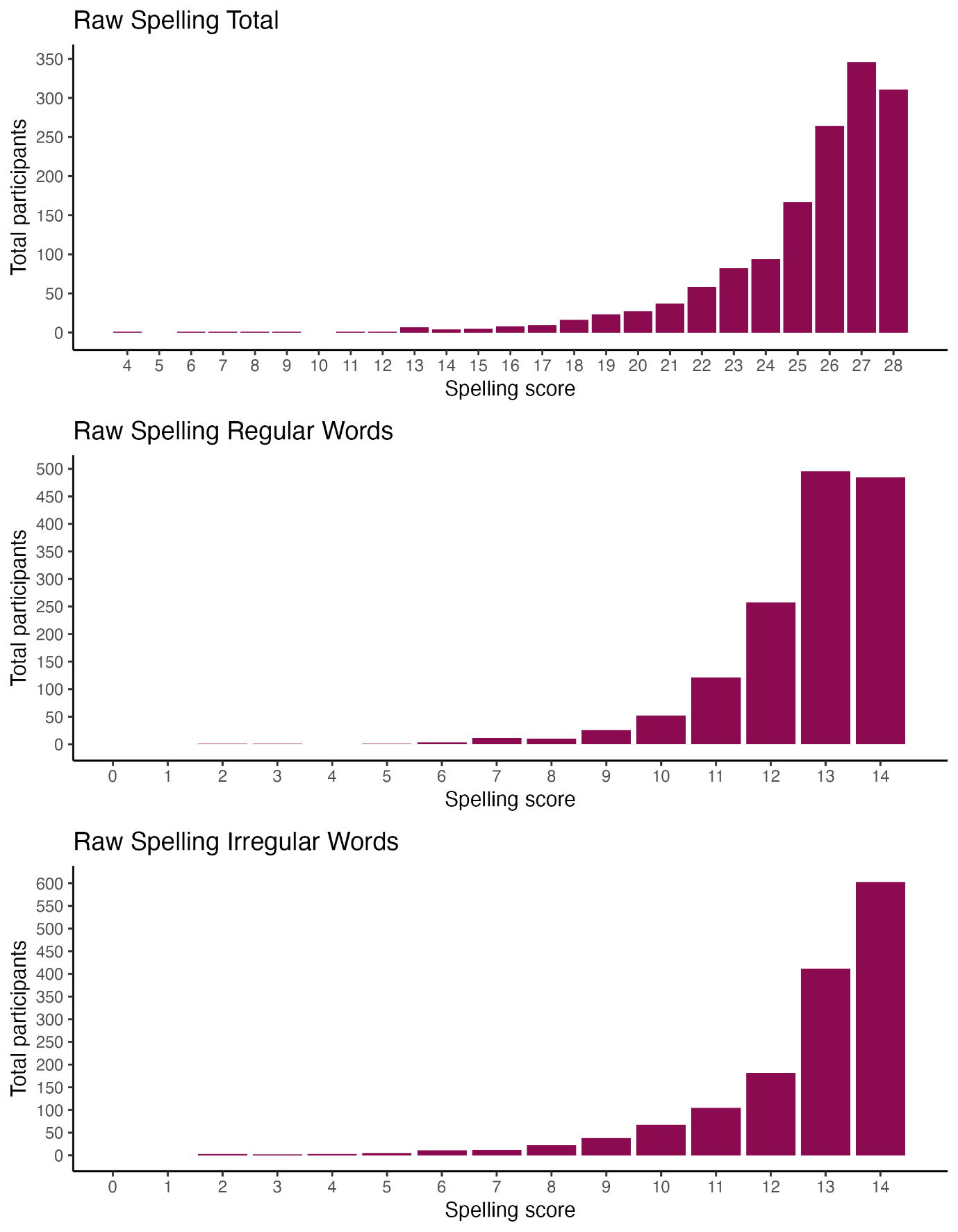


**Supplementary Figure 2:** Distributions of raw spelling scores, prior to outlier winsorisation. a) Total spelling score, b) regular words score, and c) irregular words scores.


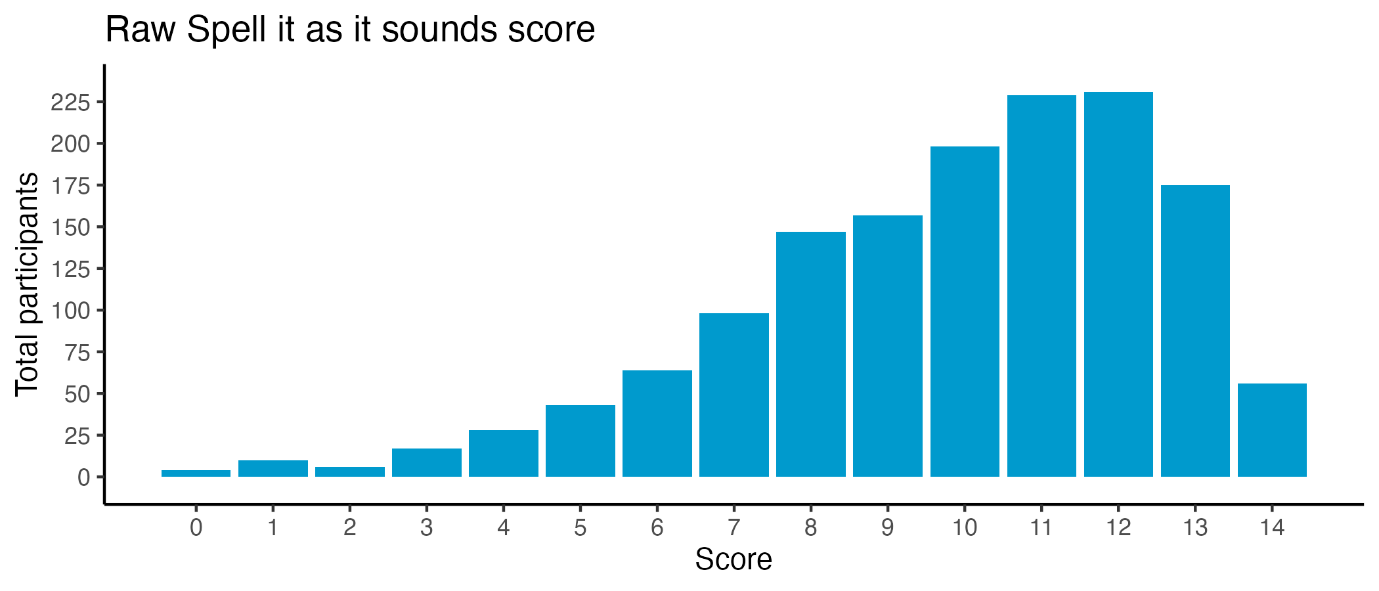


**Supplementary Figure 3:** Distributions of raw Spell it as it sounds scores, prior to outlier winsorisation.


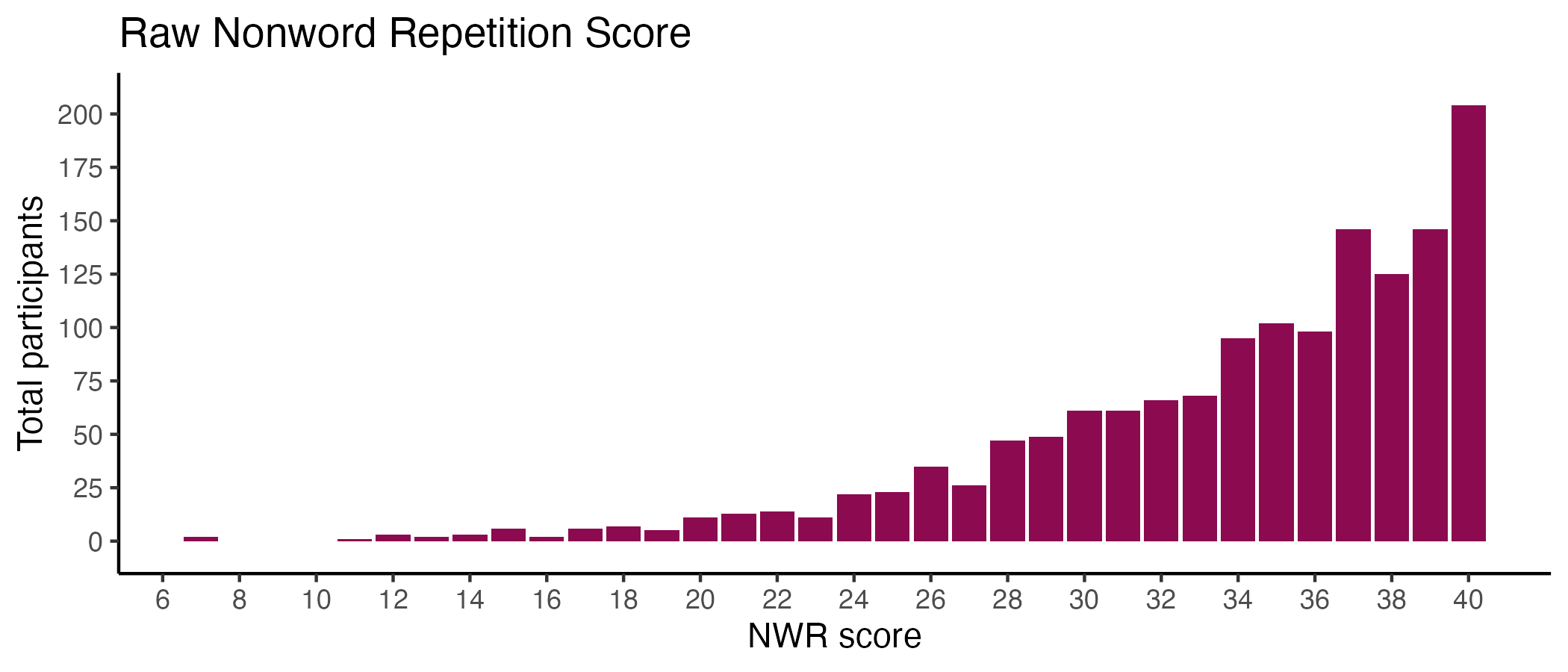


**Supplementary Figure 4:** Distributions of raw nonword repetition scores, prior to outlier winsorisation.


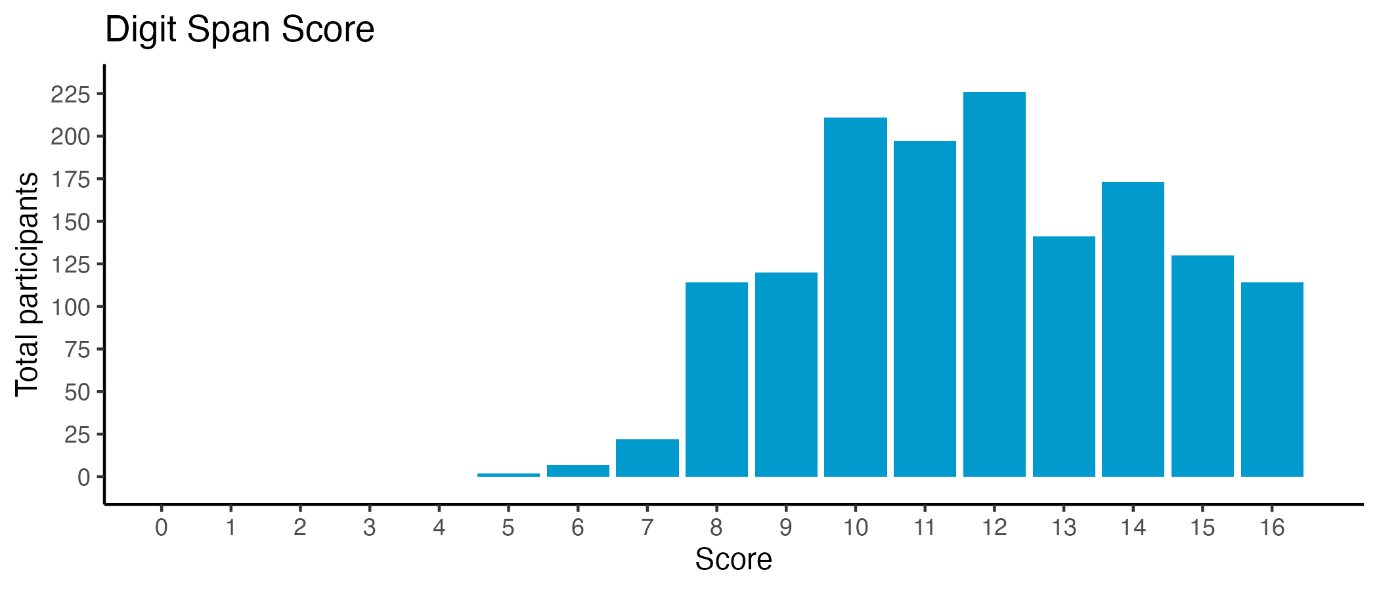


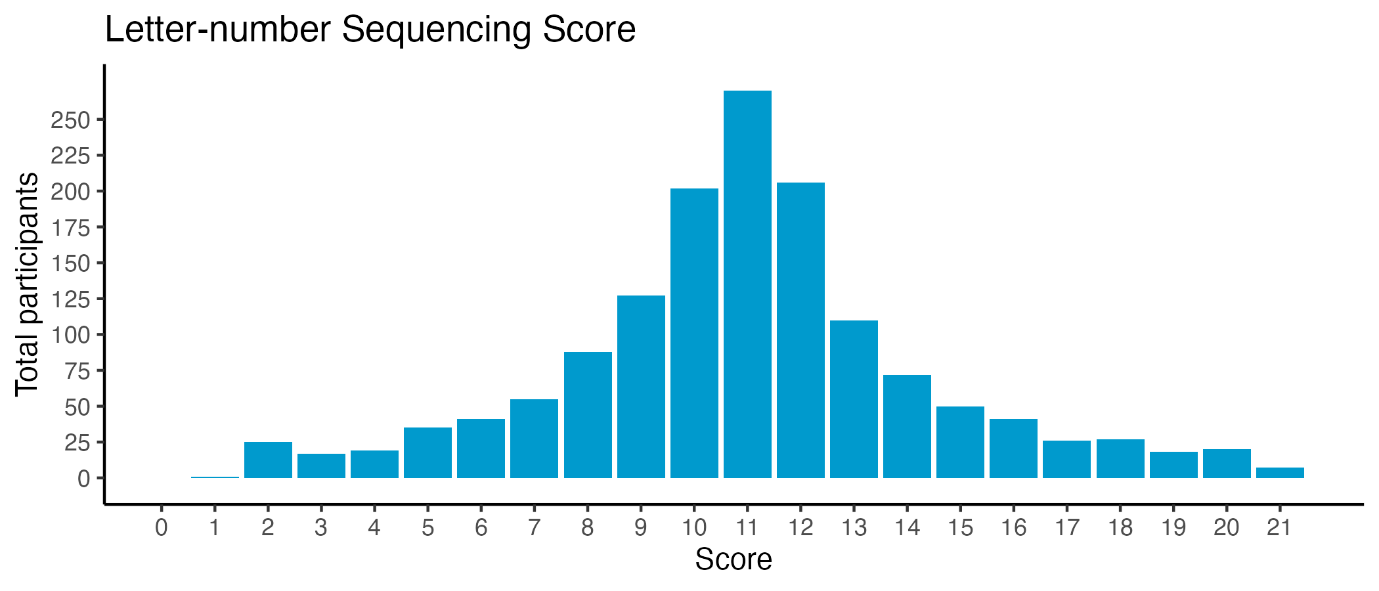


**Supplementary Figure 5:** Distributions of WAIS-III IQ auditory working memory scores prior to outlier winsorisation, of a) digit span scores and b) letter-number sequencing scores.


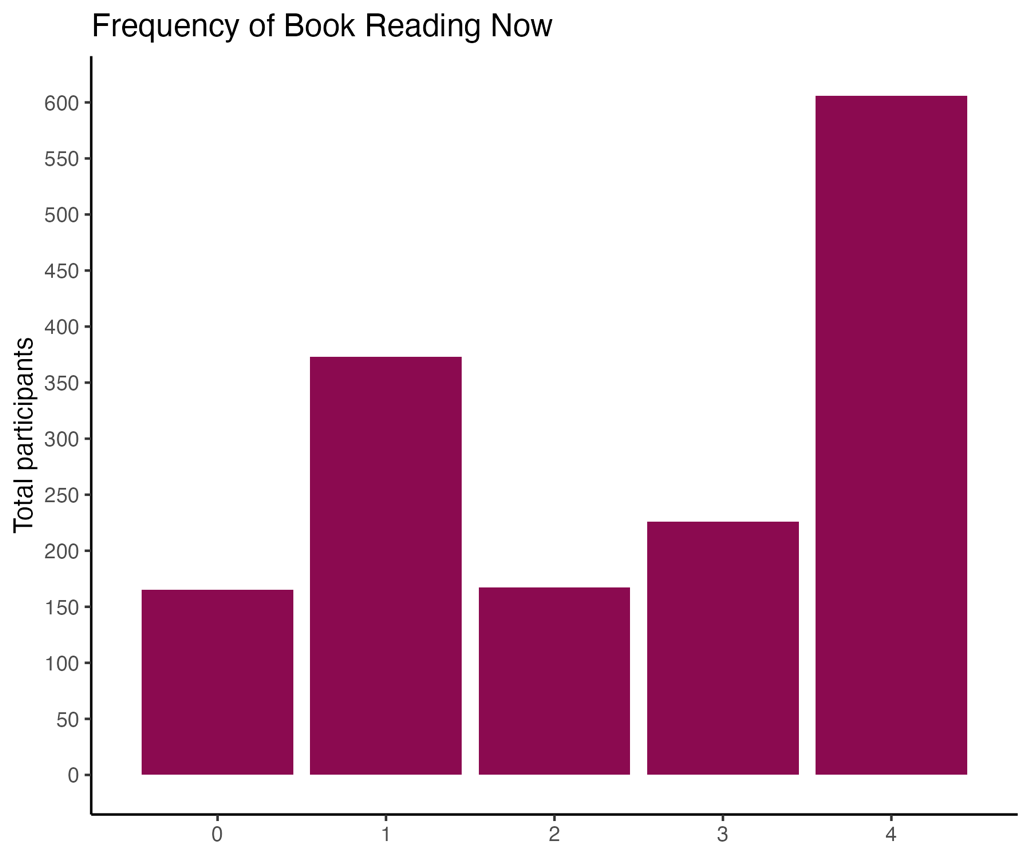


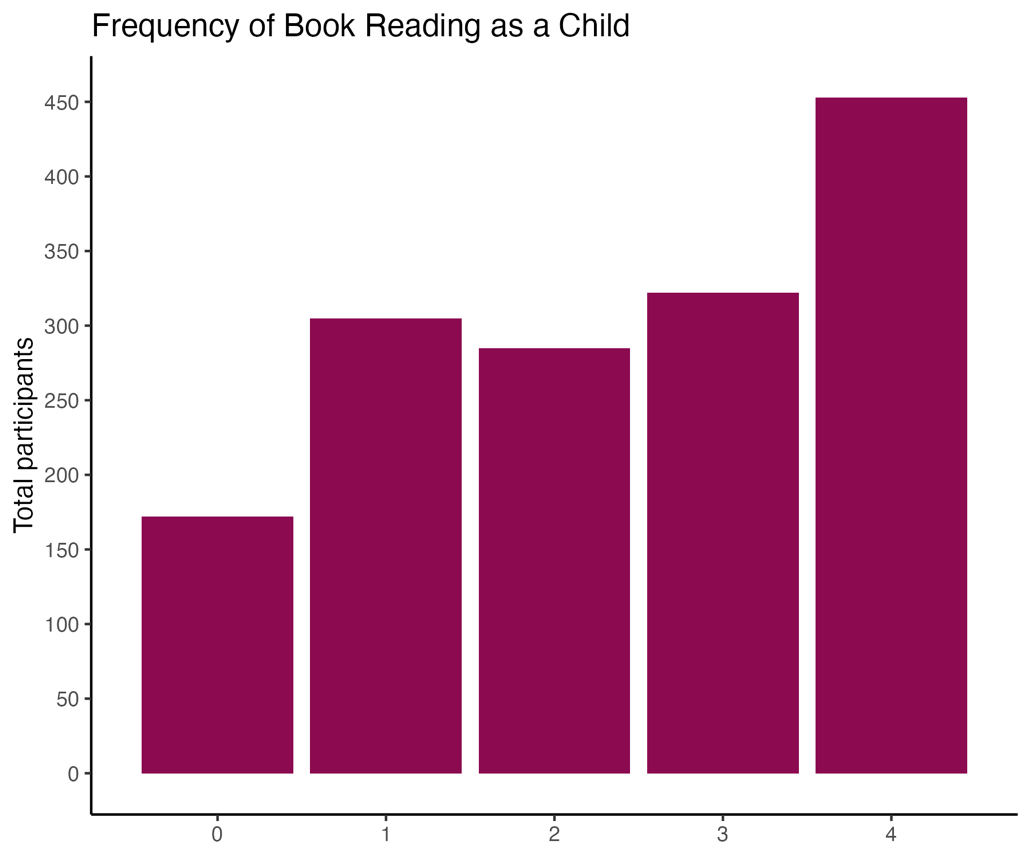


**Supplementary Figure 6:** Distributions of frequency of book reading a) now as an adult, and b) as a child.

**
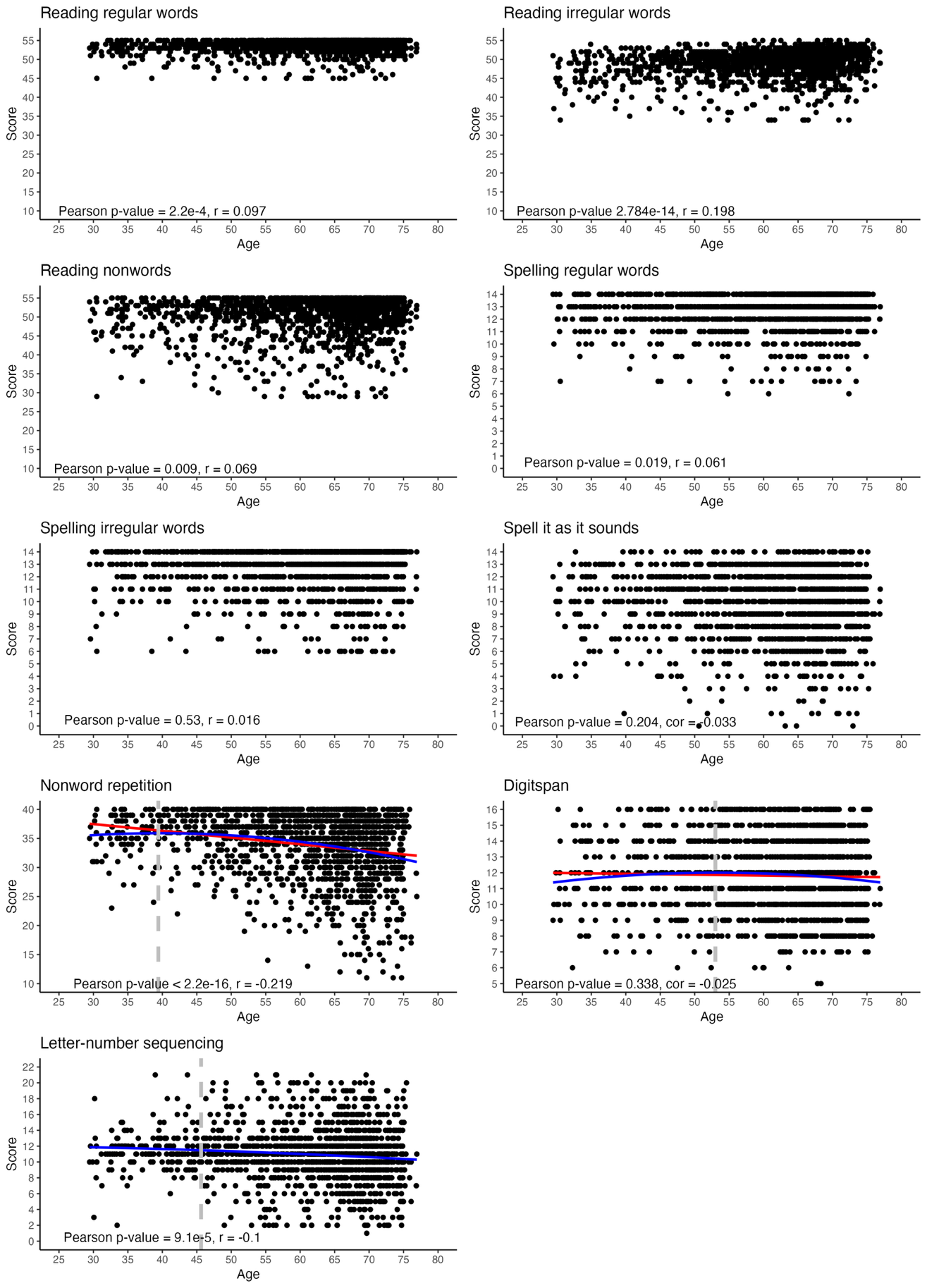
**

**Supplementary Figure 7:** Scatter plots of individual raw scores from or a) regular, b) irregular and c) nonword reading, d) regular and e) irregular spelling, f) spell it as it sounds, g) nonword repetition, h) digit span and i) letter number sequencing, by participant age at testing. Red line indicates linear regression, blue line indicates quadratic effect, and grey dashed line indicates the age of quadratic effect, presented for measures significantly associated in the multiple regression model.


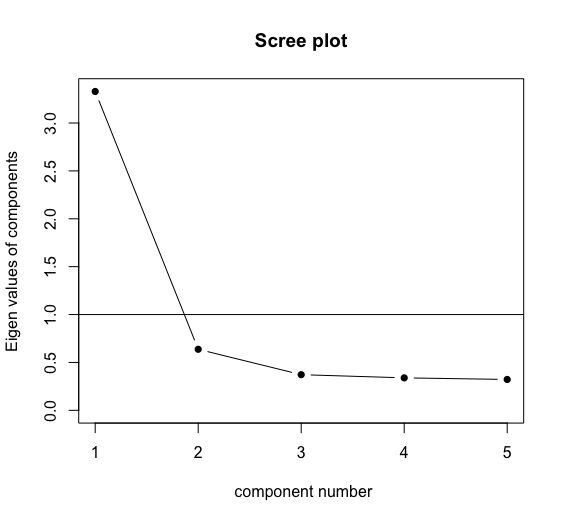


**Supplementary Figure 8:** Scree plot of eigenvalues from a principal component analysis of residualised reading and spelling measures. The first principal component explains 66.6% of the variance.


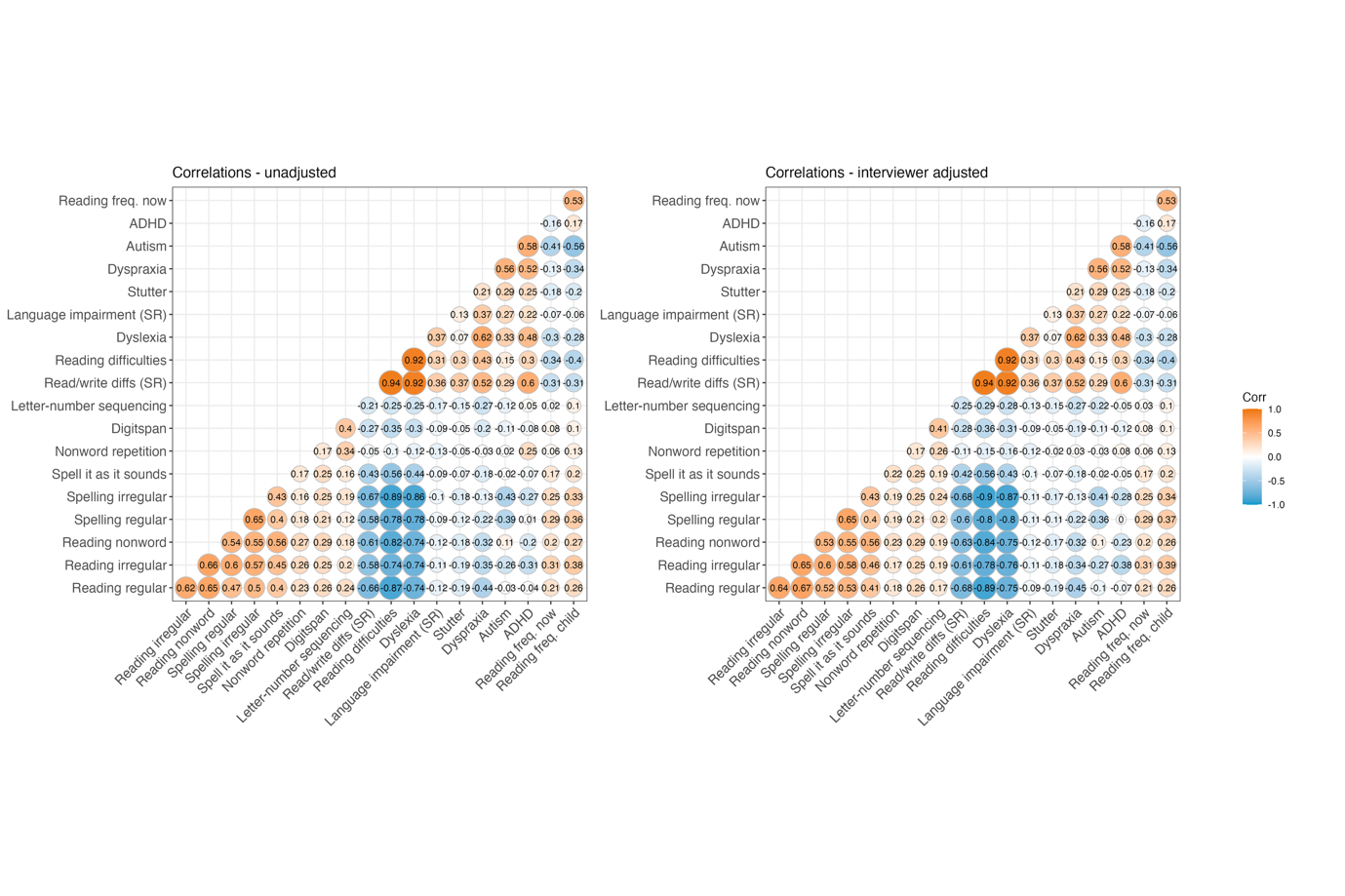


**Supplementary Figure 9:** Pearson correlations (*mixedCor*) between a) raw scores and b) interviewer adjusted scores and neurodevelopmentally relevant phenotypes.
