# Supplementary material for: Measuring reading and language skill in generation Scotland: Scottish Family Health Study
Source: BMJ Public Health. 2026 May 21;4(2):e004427. doi: 10.1136/bmjph-2025-004427 (PMC13202090; doi:10.1136/bmjph-2025-004427)
Supplement: online supplemental file 2 [file bmjph-4-2-s002.docx]

**SUPPLEMENTARY TABLES**

**Supplementary Table 1:** Descriptive statistics at the test group level after exclusions.

|  | N | Percentage female | Age range | Mean (SD) |
| --- | --- | --- | --- | --- |
| Whole cohort | 1595 | 67.5% | 29.4 - 76.9 | 60.3 (11.04) |
| All 3 tests completed | 1396 | 67.4% | 29.4 - 76.9 | 60.7 (10.8) |
| Reading (Testable) | 1454 | 67.3% | (29.4-76.9) | 60.8 (10.8) |
| Spelling, NWR, IQ, spell it as it sounds (Qualtrics) | 1465 | 67.4% | (29.4-76.9) | 60.8 (10.8) |
| Self-report questionnaire | 1537 | 67.6% | (28.4-76.7) | 60.1 (11.1) |

**Supplementary Table 2:** Multiple regression analyses to predict the effect of age, age^2^ and sex on reading, spelling, language and verbal memory measures

| **Model** | **Β** | ***SE*** | ***t*** | ***p*** | ***F*** | ***DF*** | ***P-value*** | **Adjusted *R^2^*** |
| --- | --- | --- | --- | --- | --- | --- | --- | --- |
| Regular word reading |  |  |  |  | 4.91 | 1450 | 0.002 | 0.008 |
| Intercept | 51.43 | 1.1 | 46.6 | <2x10^-16^ |  |  |  |  |
| Sex | 0.03 | 0.1 | 0.32 | 0.75 |  |  |  |  |
| Age | 0.05 | 0.04 | 1.4 | 0.17 |  |  |  |  |
| Age^2^ | -0.00 | 0.00 | -0.97 | 0.33 |  |  |  |  |
| Irregular word reading |  |  |  |  | 20.1 | 1450 | <9.1e-13 | 0.04 |
| Intercept | 42.82 | 2.25 | 19.05 | <2x10^-16^ |  |  |  |  |
| Sex | -0.1 | 0.20 | -0.5 | 0.62 |  |  |  |  |
| Age | 0.15 | 0.08 | 1.83 | 0.07 |  |  |  |  |
| Age^2^ | -0.00 | 0.00 | -1.02 | 0.31 |  |  |  |  |
| Nonword reading |  |  |  |  | 2.5 | 1450 | 0.06 | 0.003 |
| Intercept | 40.02 | 3.28 | 15.0 | <2x10^-16^ |  |  |  |  |
| Sex | 0.19 | 0.3 | 0.65 | 0.51 |  |  |  |  |
| Age | -0.01 | 0.12 | -0.1 | 0.92 |  |  |  |  |
| Age^2^ | 0.00 | 0.00 | 0.4 | 0.7 |  |  |  |  |
| Regular word spelling |  |  |  |  | 4.48 | 1461 | 0.004 | 0.007 |
| Intercept | 11.83 | 0.88 | 13.49 | <2x10^-16^ |  |  |  |  |
| Sex | 0.22 | 0.08 | 2.81 | 0.005 |  |  |  |  |
| Age | 0.01 | 0.03 | 0.24 | 0.81 |  |  |  |  |
| Age^2^ | 0.00 | 0.00 | 0.03 | 0.98 |  |  |  |  |
| Irregular word spelling |  |  |  |  | 4.1 | 1461 | 0.007 | 0.006 |
| Intercept | 10.68 | 1.08 | 9.85 | <2x10^-16^ |  |  |  |  |
| Sex | 0.31 | 0.1 | 3.2 | 0.001 |  |  |  |  |
| Age | 0.05 | 0.04 | 1.28 | 0.2 |  |  |  |  |
| Age^2^ | -0.00 | 0.00 | -1.2 | 0.23 |  |  |  |  |
| Spell it as it sounds |  |  |  |  | 0.71 | 1459 | 0.55 | -0.0006 |
| Intercept | 9.19 | 1.69 | 5.4 | 6.6x10^-8^ |  |  |  |  |
| Sex | 0.04 | 0.15 | 0.24 | 0.81 |  |  |  |  |
| Age | 0.03 | 0.06 | 0.53 | 0.6 |  |  |  |  |
| Age^2^ | -0.00 | 0.00 | -0.67 | 0.5 |  |  |  |  |
| Nonword repetition |  |  |  |  | 28.7 | 1456 | <2.2e-16 | 0.054 |
| Intercept | 29.51 | 3.48 | 8.49 | <2x10^-16^ |  |  |  |  |
| Sex | 0.45 | 0.31 | 1.46 | 0.15 |  |  |  |  |
| Age | 0.28 | 0.13 | 2.25 | 0.02 |  |  |  |  |
| Age^2^ | -0.00 | 0.00 | -3.18 | 0.002 | Quadratic effect at 39.42 years of age | | | |
| Digit span |  |  |  |  | 3.12 | 1453 | 0.03 | 0.004 |
| Intercept | 9.29 | 1.52 | 6.12 | 1.2x10^-9^ |  |  |  |  |
| Sex | -0.25 | 0.14 | -1.84 | 0.07 |  |  |  |  |
| Age | 0.12 | 0.05 | 2.15 | 0.03 |  |  |  |  |
| Age^2^ | -0.00 | 0.00 | -2.28 | 0.02 | Quadratic effect at 53.00 years of age | | | |
| Letter-number sequencing |  |  |  |  | 7.28 | 1453 | 7.6e-5 | 0.013 |
| Intercept | 7.86 | 2.16 | 3.63 | 2.9x10^-4^ |  |  |  |  |
| Sex | 0.2 | 0.19 | 1.03 | 0.3 |  |  |  |  |
| Age | 0.15 | 0.08 | 1.85 | 0.06 |  |  |  |  |
| Age^2^ | -0.00 | 0.00 | -2.28 | 0.02 | Quadratic effect at 45.61 years of age | | | |

**Supplementary Table 3**: Intercorrelations (Pearson’s r using *mixedCor*) between reading, spelling, language and verbal memory measures and discrete variables. p ≤0.05 are indicated with *. Correlations are not adjusted for case nonindependence (i.e., family relatedness).

|  | **Regular word reading** | **Irregular word reading** | **Nonword reading** | **Regular spelling** | **Irregular spelling** | **Spell it as it sounds** | **Nonword repetition** | **Digit span** | **Letter-number sequencing** | **Read/write difficulties** | **Reading difficulties** | **Dyslexia** | **Language impairment** | **Stuttering** | **Dyspraxia** | **Autism** | **ADHD** | **Developmental Delay** | **OCD** | **Depression** | **Social Anxiety Disorder** | **Eating Disorder** | **Personality Disorder** | **Bipolar Disorder** | **Reading freq. now** | **Reading freq. child** |
| --- | --- | --- | --- | --- | --- | --- | --- | --- | --- | --- | --- | --- | --- | --- | --- | --- | --- | --- | --- | --- | --- | --- | --- | --- | --- | --- |
| **Regular word reading** | 1 |  |  |  |  |  |  |  |  |  |  |  |  |  |  |  |  |  |  |  |  |  |  |  |  |  |
| **Irregular word reading** | 0.51* | 1 |  |  |  |  |  |  |  |  |  |  |  |  |  |  |  |  |  |  |  |  |  |  |  |  |
| **Nonword reading** | 0.52* | 0.59* | 1 |  |  |  |  |  |  |  |  |  |  |  |  |  |  |  |  |  |  |  |  |  |  |  |
| **Regular spelling** | 0.35* | 0.51* | 0.48* | 1 |  |  |  |  |  |  |  |  |  |  |  |  |  |  |  |  |  |  |  |  |  |  |
| **Irregular spelling** | 0.35* | 0.44* | 0.44* | 0.58* | 1 |  |  |  |  |  |  |  |  |  |  |  |  |  |  |  |  |  |  |  |  |  |
| **Spell it as it sounds** | 0.32* | 0.44* | 0.5* | 0.33* | 0.36* | 1 |  |  |  |  |  |  |  |  |  |  |  |  |  |  |  |  |  |  |  |  |
| **Nonword repetition** | 0.23* | 0.31* | 0.3* | 0.16* | 0.14* | 0.15* | 1 |  |  |  |  |  |  |  |  |  |  |  |  |  |  |  |  |  |  |  |
| **Digit span** | 0.22* | 0.25* | 0.29* | 0.2* | 0.22* | 0.24* | 0.17* | 1 |  |  |  |  |  |  |  |  |  |  |  |  |  |  |  |  |  |  |
| **Letter-number sequencing** | 0.25* | 0.22* | 0.21* | 0.14* | 0.2* | 0.17* | 0.31* | 0.41* | 1 |  |  |  |  |  |  |  |  |  |  |  |  |  |  |  |  |  |
| **Read/write difficulties** | -0.47* | -0.44* | -0.48* | -0.45* | -0.51* | -0.41* | -0.06 | -0.29* | -0.23* | 1 |  |  |  |  |  |  |  |  |  |  |  |  |  |  |  |  |
| **Reading difficulties** | -0.63* | -0.59* | -0.64* | -0.62* | -0.66* | -0.53* | -0.1 | -0.38* | -0.28* | 0.9* | 1 |  |  |  |  |  |  |  |  |  |  |  |  |  |  |  |
| **Dyslexia** | -0.55* | -0.54* | -0.57* | -0.58* | -0.61* | -0.42* | -0.14 | -0.31* | -0.27* | 0.87* | 0.86* | 1 |  |  |  |  |  |  |  |  |  |  |  |  |  |  |
| **Language impairment** | -0.13 | -0.1 | -0.09 | -0.04 | -0.04 | -0.09 | -0.17* | -0.13 | -0.19* | 0.37* | 0.3* | 0.35* | 1 |  |  |  |  |  |  |  |  |  |  |  |  |  |
| **Stuttering** | -0.1 | -0.13 | -0.07 | -0.02 | -0.09 | -0.04 | -0.04 | -0.06 | -0.14 | 0.37* | 0.3* | 0.21 | 0.2 | 1 |  |  |  |  |  |  |  |  |  |  |  |  |
| **Dyspraxia** | -0.29 | -0.24 | -0.27 | -0.13 | -0.13 | -0.2 | -0.13 | -0.22 | -0.33 | 0.5 | 0.44 | 0.59 | 0.37 | 0.2 | 1 |  |  |  |  |  |  |  |  |  |  |  |
| **Autism** | 0.07 | -0.23 | 0.32 | -0.33 | -0.32 | -0.04 | -0.09 | -0.12 | -0.14 | 0.28 | 0.16 | 0.32 | 0.27 | 0.28 | 0.56 | 1 |  |  |  |  |  |  |  |  |  |  |
| **ADHD** | 0.24 | -0.09 | -0.01 | 0.23 | 0.02 | -0.13 | 0.08 | -0.11 | 0.01 | 0.56 | 0.31 | 0.46 | 0.22 | 0.24 | 0.53 | 0.59 | 1 |  |  |  |  |  |  |  |  |  |
| **Developmental Delay** | 0.03 | -0.06 | 0.08 | -0.15 | 0.1 | 0.09 | -0.28 | -0.28 | -0.38 | 0.51 | 0.37 | 0.51 | 0.48 | 0.48 | 0.77 | 0.62 | 0.59 | 1 |  |  |  |  |  |  |  |  |
| **OCD** | 0.17 | -0.12 | -0.13 | 0.06 | 0 | -0.02 | -0.01 | -0.02 | -0.16 | 0.36 | 0.32 | 0.49 | 0.27 | 0.45 | 0.44 | 0.51 | 0.46 | 0.51 | 1 |  |  |  |  |  |  |  |
| **Depression** | 0 | -0.03 | 0 | -0.05 | -0.07 | -0.04 | -0.06 | -0.08 | -0.12* | 0.19* | 0.18* | 0.32* | 0.07 | 0.21* | 0.18 | 0.37 | 0.26 | 0.37 | 0.38 | 1 |  |  |  |  |  |  |
| **Social Anxiety Disorder** | -0.11 | -0.19* | -0.09 | -0.12 | -0.22* | -0.19* | -0.09 | -0.05 | -0.14 | 0.35* | 0.29* | 0.34* | 0.34* | 0.35* | 0.2 | 0.48 | 0.24 | 0.28 | 0.46 | 0.62* | 1 |  |  |  |  |  |
| **Eating Disorder** | 0.02 | 0.03 | 0.01 | -0.07 | 0.05 | -0.1 | -0.06 | -0.06 | -0.16* | 0.01 | -0.04 | 0.03 | 0.09 | -0.02 | 0.3 | 0.38 | 0.33 | 0.38 | 0.4 | 0.28* | 0.25 | 1 |  |  |  |  |
| **Personality Disorder** | -0.03 | -0.16 | -0.19 | -0.24 | -0.22 | -0.62 | 0.05 | -0.16 | 0.04 | 0.36 | 0.38 | 0.28 | 0.36 | 0.21 | 0.48 | 0.56 | 0.5 | 0.54 | 0.43 | 0.59 | 0.67 | 0.48 | 1 |  |  |  |
| **Bipolar Disorder** | -0.05 | -0.19 | 0.11 | -0.06 | -0.26 | -0.15 | 0.04 | -0.26 | -0.22 | 0.14 | 0.2 | 0.38 | 0.16 | 0.34 | 0.47 | 0.54 | 0.49 | 0.54 | 0.42 | 0.54 | 0.33 | 0.28 | 0.47 | 1 |  |  |
| **Reading freq. now** | 0.14* | 0.28* | 0.15* | 0.21* | 0.19* | 0.18* | 0.1* | 0.1* | 0.03 | -0.28* | -0.32* | -0.29* | -0.07 | -0.2* | -0.13 | -0.4 | -0.15 | 0.03 | -0.2 | -0.06 | -0.2* | -0.06 | -0.16 | -0.21 | 1 |  |
| **Reading freq. child** | 0.24* | 0.39* | 0.21* | 0.3* | 0.26* | 0.19* | 0.12* | 0.12* | 0.11* | -0.3* | -0.38* | -0.26* | -0.06 | -0.23* | -0.33 | -0.55 | 0.17 | -0.37 | 0.04 | -0.05 | -0.16* | -0.07 | -0.01 | -0.01 | 0.53* | 1 |

**Supplementary Table 4:** Table shows intercorrelations (Pearson’s r) calculated using *mixedCor* (*Psych*), that met statistical significance (FDR p ≤0.05) and bootstrapped confidence intervals. Correlations are not adjusted for case nonindependence (i.e., family relatedness).

| **Correlation** | **mixedCor estimate (Pearson’s r)** | **CI lower** | **CI upper** | **P** | **P FDR** |
| --- | --- | --- | --- | --- | --- |
| Reading regular-Reading irregular | 0.51 | 0.47 | 0.55 | 0 | 0 |
| Reading regular-Reading nonwords | 0.52 | 0.47 | 0.56 | 0 | 0 |
| Reading regular-Spelling regular | 0.35 | 0.31 | 0.4 | 0 | 0 |
| Reading regular-Spelling irregular | 0.35 | 0.3 | 0.39 | 0 | 0 |
| Reading regular-Spell it as it sounds | 0.32 | 0.27 | 0.36 | 0 | 0 |
| Reading regular-Nonword repetition | 0.23 | 0.18 | 0.28 | 0 | 0 |
| Reading regular-Digit span | 0.22 | 0.17 | 0.27 | 2.2x10^-16^ | 1.16x10^-15^ |
| Reading regular-Letter-number span | 0.25 | 0.21 | 0.3 | 0 | 0 |
| Reading regular-Reading or writing difficulties | -0.47 | -0.57 | -0.37 | 2.25x10^-13^ | 8.56x10^-13^ |
| Reading regular-Reading difficulties | -0.63 | -0.72 | -0.52 | 2.29x10^-13^ | 8.6x10^-13^ |
| Reading regular-Dyslexia | -0.55 | -0.68 | -0.38 | 5.00x10^-07^ | 1.15x10^-6^ |
| Reading regular-Reading freq. adult | 0.14 | 0.07 | 0.18 | 3.73x10^-06^ | 8.33x10^-6^ |
| Reading regular-Reading freq. child | 0.24 | 0.18 | 0.28 | 2.22x10^-16^ | 1.12x10^-15^ |
| Reading irregular-Reading nonwords | 0.59 | 0.55 | 0.62 | 0 | 0 |
| Reading irregular-Spelling regular | 0.51 | 0.47 | 0.55 | 0 | 0 |
| Reading irregular-Spelling irregular | 0.44 | 0.4 | 0.48 | 0 | 0 |
| Reading irregular-Spell it as it sounds | 0.44 | 0.39 | 0.48 | 0 | 0 |
| Reading irregular-Nonword repetition | 0.31 | 0.26 | 0.35 | 0 | 0 |
| Reading irregular-Digit span | 0.25 | 0.19 | 0.29 | 0 | 0 |
| Reading irregular-Letter-number span | 0.22 | 0.17 | 0.27 | 0 | 0 |
| Reading irregular-Reading or writing difficulties | -0.44 | -0.54 | -0.33 | 2.45x10^-11^ | 8.43x10^-11^ |
| Reading irregular-Reading difficulties | -0.59 | -0.68 | -0.47 | 3.64x10^-13^ | 1.33x10^-12^ |
| Reading irregular-Dyslexia | -0.54 | -0.7 | -0.34 | 3.54x10^-05^ | 7.6x10^-5^ |
| Reading irregular-Social anxiety disorder | -0.19 | -0.34 | -0.03 | 0.02 | 0.03 |
| Reading irregular-Reading freq. adult | 0.28 | 0.21 | 0.3 | 0 | 0 |
| Reading irregular-Reading freq. child | 0.39 | 0.31 | 0.41 | 0 | 0 |
| Reading nonwords-Spelling regular | 0.48 | 0.44 | 0.52 | 0 | 0 |
| Reading nonwords-Spelling irregular | 0.44 | 0.39 | 0.48 | 0 | 0 |
| Reading nonwords-Spell it as it sounds | 0.5 | 0.47 | 0.54 | 0 | 0 |
| Reading nonwords-Nonword repetition | 0.3 | 0.25 | 0.35 | 0 | 0 |
| Reading nonwords-Digit span | 0.29 | 0.24 | 0.33 | 0 | 0 |
| Reading nonwords-Letter-number span | 0.21 | 0.16 | 0.26 | 8.88x10^-16^ | 4.02x10^-15^ |
| Reading nonwords-Reading or writing difficulties | -0.48 | -0.57 | -0.38 | 1.44x10^-14^ | 6.21x10^-14^ |
| Reading nonwords-Reading difficulties | -0.64 | -0.72 | -0.54 | 2.22x10^-16^ | 1.09x10^-15^ |
| Reading nonwords-Dyslexia | -0.57 | -0.7 | -0.41 | 1.88x10^-07^ | 4.55x10^-7^ |
| Reading nonwords-Reading freq. adult | 0.15 | 0.09 | 0.19 | 2.79x10^-07^ | 6.57x10^-7^ |
| Reading nonwords-Reading freq. child | 0.21 | 0.15 | 0.25 | 1.02x10^-12^ | 3.64x10^-12^ |
| Spelling regular-Spelling irregular | 0.58 | 0.54 | 0.62 | 0 | 0 |
| Spelling regular-Spell it as it sounds | 0.33 | 0.29 | 0.37 | 0 | 0 |
| Spelling regular-Nonword repetition | 0.16 | 0.11 | 0.2 | 1.07x10^-09^ | 3.34x10^-9^ |
| Spelling regular-Digit span | 0.2 | 0.15 | 0.25 | 4.66x10^-15^ | 2.06x10^-14^ |
| Spelling regular-Letter-number span | 0.14 | 0.09 | 0.19 | 2.77x10^-08^ | 7.57x10^-8^ |
| Spelling regular-Reading or writing difficulties | -0.45 | -0.54 | -0.35 | 1.47x10^-13^ | 5.73x10^-13^ |
| Spelling regular-Reading difficulties | -0.62 | -0.7 | -0.53 | 0 | 0 |
| Spelling regular-Dyslexia | -0.58 | -0.7 | -0.43 | 5.97x10^-08^ | 1.53x10^-7^ |
| Spelling regular-Reading freq. adult | 0.21 | 0.14 | 0.24 | 8.40x10^-12^ | 2.95x10^-11^ |
| Spelling regular-Reading freq. child | 0.3 | 0.23 | 0.33 | 0 | 0 |
| Spelling irregular-Spell it as it sounds | 0.36 | 0.31 | 0.4 | 0 | 0 |
| Spelling irregular-Nonword repetition | 0.14 | 0.08 | 0.19 | 8.75x10^-07^ | 1.98x10^-6^ |
| Spelling irregular-Digit span | 0.22 | 0.17 | 0.27 | 2.22x10^-16^ | 1.06x10^-15^ |
| Spelling irregular-Letter-number span | 0.2 | 0.15 | 0.25 | 6.64x10^-14^ | 2.66x10^-13^ |
| Spelling irregular-Reading or writing difficulties | -0.51 | -0.6 | -0.41 | 2.18x10^-14^ | 8.91x10^-14^ |
| Spelling irregular-Reading difficulties | -0.66 | -0.75 | -0.56 | 2.00x10^-14^ | 8.38x10^-14^ |
| Spelling irregular-Dyslexia | -0.61 | -0.73 | -0.46 | 2.01x10^-08^ | 5.58x10^-8^ |
| Spelling irregular-Social anxiety disorder | -0.22 | -0.35 | -0.09 | 1.30x10^-03^ | 2.41x10^-3^ |
| Spelling irregular-Reading freq. adult | 0.19 | 0.12 | 0.22 | 3.56x10^-10^ | 1.3x10^-9^ |
| Spelling irregular-Reading freq. child | 0.26 | 0.19 | 0.3 | 0 | 0 |
| Spell it as it sounds-Nonword repetition | 0.15 | 0.1 | 0.2 | 3.32x10^-08^ | 8.94x10^-5^ |
| Spell it as it sounds-Digit span | 0.24 | 0.19 | 0.28 | 0 | 0 |
| Spell it as it sounds-Letter-number span | 0.17 | 0.12 | 0.22 | 1.32x10^-09^ | 4.05x10^-9^ |
| Spell it as it sounds-Reading or writing difficulties | -0.41 | -0.49 | -0.32 | 4.44x10^-16^ | 2.06x10^-15^ |
| Spell it as it sounds-Reading difficulties | -0.53 | -0.6 | -0.44 | 0 | 0 |
| Spell it as it sounds-Dyslexia | -0.42 | -0.52 | -0.3 | 1.75x10^-09^ | 5.09x10^-9^ |
| Spell it as it sounds-Social anxiety disorder | -0.19 | -0.32 | -0.05 | 0.01 | 0.02 |
| Spell it as it sounds-Reading freq. adult | 0.18 | 0.11 | 0.21 | 2.94x10^-09^ | 8.42x10^-9^ |
| Spell it as it sounds-Reading freq. child | 0.19 | 0.12 | 0.22 | 1.02x10^-10^ | 3.37x10^-10^ |
| Nonword repetition-Digit span | 0.17 | 0.12 | 0.22 | 5.37x10^-11^ | 1.81x10^-10^ |
| Nonword repetition-Letter-number span | 0.31 | 0.26 | 0.35 | 0 | 0 |
| Nonword repetition-Language impairment | -0.17 | -0.29 | -0.04 | 9.15x10^-03^ | 0.02 |
| Nonword repetition-Reading freq. adult | 0.1 | 0.04 | 0.15 | 8.03x10^-04^ | 1.55x10^-3^ |
| Nonword repetition-Reading freq. child | 0.12 | 0.07 | 0.17 | 1.01x10^-05^ | 2.22x10^-5^ |
| Digit span-Letter-number span | 0.41 | 0.36 | 0.45 | 0 | 0 |
| Digit span-Reading or writing difficulties | -0.29 | -0.39 | -0.19 | 6.32x10^-08^ | 1.6x10^-7^ |
| Digit span-Reading difficulties | -0.38 | -0.48 | -0.27 | 1.48x10^-09^ | 4.39x10^-9^ |
| Digit span-Dyslexia | -0.31 | -0.47 | -0.14 | 8.56x10^-04^ | 1.62x10^-3^ |
| Digit span-Reading freq. adult | 0.1 | 0.03 | 0.14 | 1.6x10^-03^ | 2.89x10^-3^ |
| Digit span-Reading freq. child | 0.12 | 0.06 | 0.16 | 1.79x10^-05^ | 3.91x10^-5^ |
| Letter-number span-Reading or writing difficulties | -0.23 | -0.32 | -0.15 | 3.46x10^-07^ | 8.04x10^-7^ |
| Letter-number span-Reading difficulties | -0.28 | -0.37 | -0.18 | 1.54x10^-07^ | 3.84x10^-7^ |
| Letter-number span-Dyslexia | -0.27 | -0.41 | -0.12 | 5.77x10^-04^ | 1.14x10^-3^ |
| Letter-number span-Language impairment | -0.19 | -0.27 | -0.09 | 1.75x10^-03^ | 2.06x10^-3^ |
| Letter-number span-Depression | -0.12 | -0.19 | -0.05 | 1.75x10^-04^ | 2.06x10^-3^ |
| Letter-number span-Eating disorder | -0.16 | -0.28 | -0.03 | 0.01660354 | 0.03 |
| Letter-number span-Reading freq. child | 0.11 | 0.05 | 0.15 | 1.08x10^-04^ | 2.3x10^-4^ |
| Reading or writing difficulties-Reading difficulties | 0.9 | 0.93 | 0.99 | 1.11x10^-04^ | 2.32x10^-3^ |
| Reading or writing difficulties-Dyslexia | 0.87 | 0.85 | 0.95 | 1.18x10^-10^ | 3.84x10^-10^ |
| Reading or writing difficulties-Language impairment | 0.37 | 0.19 | 0.53 | 2.56x10^-04^ | 5.24x10^-4^ |
| Reading or writing difficulties-Stuttering | 0.37 | 0.19 | 0.53 | 3.09x10^-04^ | 6.256x10^-4^ |
| Reading or writing difficulties-Depression | 0.19 | 0.09 | 0.31 | 8.47x10^-04^ | 1.23x10^-3^ |
| Reading or writing difficulties-Social anxiety disorder | 0.35 | 0.18 | 0.53 | 4.29x10^-04^ | 8.57x10^-4^ |
| Reading or writing difficulties-Reading freq. adult | -0.28 | -0.34 | -0.17 | 4.73x10^-08^ | 1.23x10^-7^ |
| Reading or writing difficulties-Reading freq. child | -0.3 | -0.39 | -0.2 | 3.88x10^-08^ | 1.03x10^-7^ |
| Reading difficulties-Dyslexia | 0.86 | 0.88 | 0.97 | 1.58x10^-07^ | 3.88x10^-7^ |
| Reading difficulties-Language impairment | 0.3 | 0.07 | 0.48 | 0.01 | 0.02 |
| Reading difficulties-Stuttering | 0.3 | 0.1 | 0.49 | 4.85x10^-03^ | 8.6x10^-3^ |
| Reading difficulties-Depression | 0.18 | 0.03 | 0.3 | 0.02 | 0.03 |
| Reading difficulties-Social anxiety disorder | 0.29 | 0.04 | 0.47 | 0.03 | 0.04 |
| Reading difficulties-Reading freq. adult | -0.32 | -0.4 | -0.19 | 1.88x10^-07^ | 4.5x10^-7^ |
| Reading difficulties-Reading freq. child | -0.38 | -0.48 | -0.26 | 4.01x10^-09^ | 1.13x10^-8^ |
| Dyslexia-Language impairment | 0.35 | 0.05 | 0.56 | 0.03 | 0.04 |
| Dyslexia-Depression | 0.32 | 0.15 | 0.49 | 7.78x10^-04^ | 1.52x10^-3^ |
| Dyslexia-Social anxiety disorder | 0.34 | 0.07 | 0.59 | 0.02 | 0.03 |
| Dyslexia-Reading freq. adult | -0.29 | -0.42 | -0.11 | 1.51x10^-03^ | 2.78x10^-3^ |
| Dyslexia-Reading freq. child | -0.26 | -0.43 | -0.08 | 6.03x10^-03^ | 0.01 |
| Language impairment-Social anxiety disorder | 0.34 | 0.05 | 0.54 | 0.02 | 0.04 |
| Stuttering-Depression | 0.21 | 0.05 | 0.37 | 0.01 | 0.03 |
| Stuttering-Social anxiety disorder | 0.35 | 0.08 | 0.56 | 0.02 | 0.02 |
| Stuttering-Reading freq. adult | -0.2 | -0.31 | -0.04 | 0.01 | 0.02 |
| Stuttering-Reading freq. child | -0.23 | -0.34 | -0.08 | 2.26x10^-03^ | 4.05x10^-3^ |
| Depression-Eating disorder | 0.62 | 0.06 | 0.47 | 0.02 | 0.03 |
| Depression-Social anxiety disorder | 0.28 | 0.48 | 0.73 | 1.35x10^-09^ | 4.07x10^-9^ |
| Social anxiety disorder-Reading freq. adult | -0.2 | -0.32 | -0.04 | 0.01 | 0.02 |
| Social anxiety disorder-Reading freq. child | -0.16 | -0.3 | -0.02 | 0.03 | 0.05 |
| Reading freq. adult-Reading freq. child | 0.53 | 0.44 | 0.53 | 0 | 0 |

**Supplementary Table 5:** Factor loadings from the principal component analysis of residuals of reading and spelling measures adjusted for sex, age and age^2^ used to generate the composite reading measure (N = 1466).

| Loadings: |  |
| --- | --- |
|  | PC1 |
| Reading regular words | 0.792 |
| Reading irregular words | 0.854 |
| Reading nonwords | 0.836 |
| Spelling regular words | 0.795 |
| Spelling irregular words | 0.801 |
|  |  |
| SS loadings | 3.329 |
| Proportion Var | 0.666 |

**Supplementary Table 6:** Test for association between diagnosis and quantitative measures using Welch's two sample t-test. *p ≤ 0.05, **p ≤ 0.01, ***p≤ 0.001. *Note*: quantitative scores were scaled to a mean of 0.5 for all samples (cases and controls).

| ***Binary*** | ***Measure*** | ***t*** | ***Df*** | ***Cohens d*** | ***P Unadjusted*** | ***P FDR adjusted*** | ***control mean*** | ***case mean*** | ***95% CI*** |
| --- | --- | --- | --- | --- | --- | --- | --- | --- | --- |
| Reading/ writing difficulties | Reading composite | 11.78 | 147.51 | 1.21 | <2.2x10^-16^ | 4.13x10^-15^*** | 0.52 | 0.35 | 0.14-0.2 |
|  | Reading regular | 8.66 | 142.36 | 0.97 | 9.12x10^-15^ | 1.05x10^-13^*** | 0.51 | 0.38 | 0.11-0.17 |
|  | Reading irregular | 7.96 | 141.76 | 0.91 | 5.13x10^-13^ | 5.5x10^-12^*** | 0.51 | 0.38 | 0.1-0.16 |
|  | Reading nonwords | 9.22 | 144.47 | 0.99 | 3.37x10^-16^ | 4.6x10^-15^*** | 0.51 | 0.37 | 0.11-0.17 |
|  | Spelling regular | 8.81 | 146.76 | 0.92 | 3.18x10^-15^ | 3.97x10^-14^*** | 0.51 | 0.38 | 0.1-0.16 |
|  | Spelling irregular | 10.03 | 146.29 | 1.05 | <2.2x10^-16^ | 6.6x10^-15^ | 0.51 | 0.37 | 0.12-0.18 |
|  | Spell it as it sounds | 9.21 | 154.9 | 0.84 | 2.24x10^-16^ | 3.36x10^-15^*** | 0.51 | 0.39 | 0.09-0.15 |
|  | Nonword repetition | 1.24 | 147.61 | 0.12 | 0.22 | 0.49 | 0.5 | 0.48 | -0.01-0.05 |
|  | Digit span | 5.66 | 147.36 | 0.51 | 7.72x10^-8^ | 5.26x10^-7^*** | 0.51 | 0.42 | 0.06-0.11 |
|  | Letter-number sequencing | 5.24 | 157.44 | 0.46 | 5.13x10^-7^ | 3.21x10^-6^*** | 0.51 | 0.44 | 0.04-0.09 |
| Reading difficulties | Reading composite | 18.44 | 107.03 | 1.72 | <2.2x10^-16^ | 3.67x10^-15^*** | 0.52 | 0.28 | 0.21-0.26 |
|  | Reading regular | 11.54 | 99.02 | 1.39 | <2.2x10^-16^ | 3.3x10^-14^*** | 0.51 | 0.32 | 0.16-0.23 |
|  | Reading irregular | 10.38 | 98.31 | 1.29 | <2.2x10^-16^ | 1.65x10^-14^*** | 0.51 | 0.33 | 0.15-0.22 |
|  | Reading nonwords | 12.77 | 101.21 | 1.41 | <2.2x10^-16^ | 1.1x10^-14^*** | 0.51 | 0.32 | 0.17-0.23 |
|  | Spelling regular | 13.23 | 103.2 | 1.37 | <2.2x10^-16^ | 8.25x10^-15^*** | 0.51 | 0.32 | 0.16-0.22 |
|  | Spelling irregular | 12.97 | 100.25 | 1.48 | <2.2x10^-16^ | 5.5x10^-15^*** | 0.51 | 0.31 | 0.17-0.24 |
|  | Spell it as it sounds | 12.3 | 107.3 | 1.14 | <2.2x10^-16^ | 4.71x10^-15^*** | 0.51 | 0.35 | 0.14-0.19 |
|  | Nonword repetition | 1.72 | 98.69 | 0.21 | 0.09 | 0.28 | 0.5 | 0.47 | 0-0.07 |
|  | Digit span | 6.69 | 99.15 | 0.8 | 1.32x10^-9^ | 1.16x10^-8^*** | 0.51 | 0.39 | 0.08-0.15 |
|  | Letter-number sequencing | 5.66 | 103.12 | 0.59 | 1.37x10^-7^ | 8.92x10^-7^*** | 0.51 | 0.42 | 0.06-0.12 |
| Dyslexia | Reading composite | 9.87 | 30.5 | 1.68 | 5.26x10^-11^ | 5.26x10^-10^*** | 0.51 | 0.27 | 0.19-0.29 |
|  | Reading regular | 7.15 | 30.21 | 1.36 | 5.61x10^-8^ | 4.01x10^-7^*** | 0.51 | 0.31 | 0.14-0.25 |
|  | Reading irregular | 5.59 | 29.74 | 1.35 | 4.48x10^-6^ | 2.69x10^-5^*** | 0.51 | 0.31 | 0.12-0.27 |
|  | Reading nonwords | 7.5 | 30.21 | 1.42 | 2.23x10^-8^ | 1.76x10^-7^*** | 0.51 | 0.3 | 0.15-0.26 |
|  | Spelling regular | 7.84 | 30.28 | 1.44 | 8.83x10^-9^ | 7.36x10^-8^*** | 0.51 | 0.3 | 0.15-0.26 |
|  | Spelling irregular | 8.74 | 30.43 | 1.53 | 8.5x10^-10^ | 7.97x10^-9^*** | 0.51 | 0.29 | 0.17-0.27 |
|  | Spell it as it sounds | 7.2 | 31.18 | 1.03 | 4.09x10^-8^ | 3.07x10^-7^*** | 0.5 | 0.35 | 0.11-0.19 |
|  | Nonword repetition | 1.71 | 30.01 | 0.35 | 0.1 | 0.30 | 0.5 | 0.45 | -0.01-0.11 |
|  | Digit span | 3.57 | 29.94 | 0.76 | 1.2x10^-3^ | 6.3x10^-03^** | 0.5 | 0.39 | 0.05-0.18 |
|  | Letter-number sequencing | 3.77 | 30.38 | 0.67 | 7.0x10^-4^ | 5.09x10^-3^** | 0.5 | 0.4 | 0.05-0.15 |
| Language impairment | Reading composite | 1.68 | 45.51 | 0.27 | 0.1 | 0.30 | 0.5 | 0.46 | -0.01-0.09 |
|  | Reading regular | 1.96 | 43.73 | 0.3 | 0.06 | 0.20 | 0.5 | 0.46 | 0-0.09 |
|  | Reading irregular | 1.43 | 43.43 | 0.23 | 0.16 | 0.41 | 0.5 | 0.47 | -0.01-0.08 |
|  | Reading nonwords | 1.09 | 42.75 | 0.21 | 0.28 | 0.51 | 0.5 | 0.47 | -0.03-0.09 |
|  | Spelling regular | 0.53 | 45.16 | 0.09 | 0.6 | 0.76 | 0.5 | 0.49 | -0.04-0.06 |
|  | Spelling irregular | 0.49 | 44.84 | 0.09 | 0.63 | 0.79 | 0.5 | 0.49 | -0.04-0.07 |
|  | Spell it as it sounds | 1.26 | 45.61 | 0.2 | 0.22 | 0.5 | 0.5 | 0.47 | -0.02-0.08 |
|  | Nonword repetition | 2.77 | 45.15 | 0.4 | 0.01 | 0.04* | 0.5 | 0.44 | 0.02-0.1 |
|  | Digit span | 1.48 | 44.64 | 0.29 | 0.15 | 0.40 | 0.5 | 0.46 | -0.02-0.1 |
|  | Letter-number sequencing | 3.98 | 49.06 | 0.43 | 2.27x10^-4^ | 1.13x10^-3^** | 0.5 | 0.44 | 0.03-0.09 |
| Stuttering/ stammer | Reading composite | 1.26 | 36.06 | 0.28 | 0.22 | 0.49 | 0.5 | 0.46 | -0.02-0.11 |
|  | Reading regular | 1.16 | 36.14 | 0.25 | 0.25 | 0.49 | 0.5 | 0.47 | -0.03-0.1 |
|  | Reading irregular | 1.49 | 36.12 | 0.32 | 0.14 | 0.40 | 0.5 | 0.46 | -0.02-0.11 |
|  | Reading nonwords | 0.76 | 35.99 | 0.17 | 0.45 | 0.69 | 0.5 | 0.48 | -0.04-0.09 |
|  | Spelling regular | 0.26 | 36.09 | 0.06 | 0.8 | 0.89 | 0.5 | 0.49 | -0.06-0.07 |
|  | Spelling irregular | 1.14 | 36.39 | 0.22 | 0.26 | 0.49 | 0.5 | 0.47 | -0.03-0.09 |
|  | Spell it as it sounds | 0.52 | 36.3 | 0.1 | 0.61 | 0.76 | 0.5 | 0.49 | -0.04-0.08 |
|  | Nonword repetition | 0.48 | 36.6 | 0.09 | 0.64 | 0.79 | 0.5 | 0.49 | -0.04-0.07 |
|  | Digit span | 0.69 | 36.37 | 0.14 | 0.49 | 0.70 | 0.5 | 0.48 | -0.04-0.08 |
|  | Letter-number sequencing | 2.02 | 36.9 | 0.34 | 0.05 | 0.19 | 0.5 | 0.45 | 0-0.1 |
| Dyspraxia | Reading composite | 1.15 | 4.02 | 0.7 | 0.31 | 0.54 | 0.5 | 0.4 | -0.14-0.35 |
|  | Reading regular | 1.74 | 4.02 | 0.86 | 0.16 | 0.41 | 0.5 | 0.38 | -0.07-0.33 |
|  | Reading irregular | 1.3 | 4.02 | 0.72 | 0.26 | 0.49 | 0.5 | 0.4 | -0.12-0.33 |
|  | Reading nonwords | 1.77 | 4.03 | 0.82 | 0.15 | 0.40 | 0.5 | 0.38 | -0.07-0.31 |
|  | Spelling regular | 0.67 | 4.02 | 0.38 | 0.54 | 0.72 | 0.5 | 0.45 | -0.17-0.28 |
|  | Spelling irregular | 0.78 | 4.02 | 0.38 | 0.48 | 0.72 | 0.5 | 0.45 | -0.14-0.25 |
|  | Spell it as it sounds | 1.6 | 4.04 | 0.61 | 0.18 | 0.45 | 0.5 | 0.41 | -0.07-0.25 |
|  | Nonword repetition | 1.1 | 4.05 | 0.4 | 0.33 | 0.56 | 0.5 | 0.44 | -0.09-0.2 |
|  | Digit span | 1.13 | 4.02 | 0.65 | 0.32 | 0.55 | 0.5 | 0.41 | -0.14-0.33 |
|  | Letter-number sequencing | 5.77 | 4.2 | 1.01 | 3.87x10^-4^ | 0.02* | 0.5 | 0.35 | 0.08-0.22 |
| Autism | Reading composite | 1.63 | 2.01 | 0.75 | 0.24 | 0.49 | 0.5 | 0.39 | -0.18-0.4 |
|  | Reading regular | -0.24 | 2 | 0.21 | 0.83 | 0.92 | 0.5 | 0.53 | -0.56-0.5 |
|  | Reading irregular | 1.59 | 2.01 | 0.71 | 0.25 | 0.48 | 0.5 | 0.4 | -0.18-0.38 |
|  | Reading nonwords | -1.04 | 2 | 0.99 | 0.41 | 0.64 | 0.5 | 0.65 | -0.75-0.45 |
|  | Spelling regular | 3.47 | 2.03 | 1.04 | 0.07 | 0.24 | 0.5 | 0.35 | -0.03-0.34 |
|  | Spelling irregular | 1.86 | 2.01 | 1.02 | 0.2 | 0.48 | 0.5 | 0.35 | -0.19-0.49 |
|  | Spell it as it sounds | 0.17 | 2.01 | 0.11 | 0.88 | 0.95 | 0.5 | 0.48 | -0.41-0.45 |
|  | Nonword repetition | 0.84 | 2.02 | 0.3 | 0.49 | 0.70 | 0.5 | 0.46 | -0.17-0.26 |
|  | Digit span | 0.78 | 2.01 | 0.37 | 0.52 | 0.70 | 0.5 | 0.45 | -0.24-0.35 |
|  | Letter-number sequencing | 0.73 | 2.01 | 0.44 | 0.54 | 0.72 | 0.5 | 0.44 | -0.32-0.45 |
| ADHD | Reading composite | -0.31 | 3 | 0.35 | 0.78 | 0.91 | 0.5 | 0.55 | -0.57-0.47 |
|  | Reading regular | -0.68 | 3 | 0.73 | 0.54 | 0.72 | 0.5 | 0.61 | -0.61-0.39 |
|  | Reading irregular | 0.31 | 3.01 | 0.26 | 0.77 | 0.91 | 0.5 | 0.46 | -0.35-0.43 |
|  | Reading nonwords | 0.03 | 3 | 0.03 | 0.98 | 0.99 | 0.5 | 0.5 | -0.46-0.47 |
|  | Spelling regular | -0.78 | 3.01 | 0.7 | 0.49 | 0.70 | 0.5 | 0.6 | -0.52-0.31 |
|  | Spelling irregular | -0.05 | 3 | 0.06 | 0.96 | 0.99 | 0.5 | 0.51 | -0.52-0.5 |
|  | Spell it as it sounds | 0.94 | 3.02 | 0.39 | 0.42 | 0.64 | 0.5 | 0.44 | -0.14-0.25 |
|  | Nonword repetition | -1.69 | 3.22 | 0.25 | 0.18 | 0.46 | 0.5 | 0.54 | -0.1-0.03 |
|  | Digit span | 0.42 | 3.01 | 0.32 | 0.7 | 0.86 | 0.5 | 0.45 | -0.31-0.41 |
|  | Letter-number sequencing | -0.04 | 3.02 | 0.02 | 0.97 | 0.99 | 0.5 | 0.5 | -0.22-0.21 |
| Developmental Delay | Reading composite | 0.21 | 2.05 | 0.05 | 0.85 | 0.93 | 0.5 | 0.5 | -0.14-0.15 |
|  | Reading regular | -1.19 | 2.49 | 0.1 | 0.34 | 0.55 | 0.5 | 0.52 | -0.06-0.03 |
|  | Reading irregular | 0.37 | 2.01 | 0.17 | 0.75 | 0.89 | 0.5 | 0.48 | -0.27-0.32 |
|  | Reading nonwords | -0.41 | 2.01 | 0.24 | 0.72 | 0.87 | 0.5 | 0.54 | -0.4-0.33 |
|  | Spelling regular | 1.85 | 2.05 | 0.47 | 0.2 | 0.48 | 0.5 | 0.43 | -0.09-0.22 |
|  | Spelling irregular | -3.24 | 2.35 | 0.31 | 0.07 | 0.23 | 0.5 | 0.55 | -0.1-0.01 |
|  | Spell it as it sounds | -1.41 | 2.07 | 0.28 | 0.29 | 0.52 | 0.5 | 0.54 | -0.17-0.08 |
|  | Nonword repetition | 1.77 | 2.01 | 0.88 | 0.22 | 0.48 | 0.5 | 0.37 | -0.18-0.44 |
|  | Digit span | 0.82 | 2 | 0.88 | 0.5 | 0.70 | 0.5 | 0.37 | -0.55-0.81 |
|  | Letter-number sequencing | 3.78 | 2.03 | 1.21 | 0.06 | 0.22 | 0.5 | 0.32 | -0.02-0.38 |
| OCD | Reading composite | 0.98 | 6.13 | 0.26 | 0.36 | 0.59 | 0.5 | 0.47 | -0.06-0.13 |
|  | Reading regular | -1.05 | 6.04 | 0.5 | 0.33 | 0.56 | 0.5 | 0.58 | -0.25-0.1 |
|  | Reading irregular | 0.77 | 6.04 | 0.36 | 0.47 | 0.71 | 0.5 | 0.45 | -0.11-0.22 |
|  | Reading nonwords | 1.81 | 6.2 | 0.37 | 0.12 | 0.34 | 0.5 | 0.45 | -0.02-0.13 |
|  | Spelling regular | -0.36 | 6.04 | 0.17 | 0.73 | 0.87 | 0.5 | 0.53 | -0.19-0.14 |
|  | Spelling irregular | 0.03 | 6.06 | 0.01 | 0.97 | 0.99 | 0.5 | 0.5 | -0.14-0.14 |
|  | Spell it as it sounds | 0.2 | 6.13 | 0.05 | 0.85 | 0.93 | 0.5 | 0.49 | -0.09-0.1 |
|  | Nonword repetition | 0.06 | 6.1 | 0.02 | 0.96 | 0.99 | 0.5 | 0.5 | -0.1-0.11 |
|  | Digit span | 0.28 | 6.28 | 0.05 | 0.79 | 0.90 | 0.5 | 0.49 | -0.06-0.07 |
|  | Letter-number sequencing | 0.92 | 6.03 | 0.46 | 0.39 | 0.62 | 0.5 | 0.43 | -0.11-0.25 |
| Depression | Reading composite | 1.23 | 458.91 | 0.08 | 0.22 | 0.47 | 0.5 | 0.49 | -0.01-0.03 |
|  | Reading regular | -0.08 | 453.18 | 0.01 | 0.93 | 0.99 | 0.5 | 0.5 | -0.02-0.02 |
|  | Reading irregular | 0.67 | 455.05 | 0.04 | 0.51 | 0.69 | 0.5 | 0.5 | -0.01-0.03 |
|  | Reading nonwords | -0.01 | 457.77 | 0.00 | 0.99 | 0.99 | 0.5 | 0.5 | -0.02-0.02 |
|  | Spelling regular | 1.34 | 442.52 | 0.09 | 0.18 | 0.46 | 0.5 | 0.49 | -0.01-0.03 |
|  | Spelling irregular | 1.94 | 467.65 | 0.13 | 0.05 | 0.19 | 0.51 | 0.49 | 0-0.04 |
|  | Spell it as it sounds | 1.15 | 474.12 | 0.07 | 0.25 | 0.49 | 0.5 | 0.49 | -0.01-0.03 |
|  | Nonword repetition | 1.72 | 493.06 | 0.11 | 0.09 | 0.28 | 0.5 | 0.49 | 0-0.03 |
|  | Digit span | 2.08 | 495.21 | 0.13 | 0.04 | 0.15 | 0.51 | 0.49 | 0-0.04 |
|  | Letter-number sequencing | 3.33 | 483.66 | 0.21 | 9.49x10^-4^ | 5.09x10^-3^** | 0.51 | 0.48 | 0.01-0.05 |
| Social Anxiety Disorder | Reading composite | 2.53 | 40.95 | 0.44 | 0.02 | 0.07 | 0.5 | 0.44 | 0.01-0.12 |
|  | Reading regular | 1.25 | 39.38 | 0.25 | 0.22 | 0.48 | 0.5 | 0.47 | -0.02-0.1 |
|  | Reading irregular | 2.38 | 39.61 | 0.45 | 0.02 | 0.09 | 0.5 | 0.44 | 0.01-0.12 |
|  | Reading nonwords | 1.17 | 39.7 | 0.21 | 0.25 | 0.50 | 0.5 | 0.47 | -0.02-0.09 |
|  | Spelling regular | 1.65 | 41.13 | 0.27 | 0.11 | 0.32 | 0.5 | 0.46 | -0.01-0.09 |
|  | Spelling irregular | 3.47 | 41.58 | 0.53 | 1.23x10^-3^ | 6.15x10^-3^** | 0.5 | 0.43 | 0.03-0.12 |
|  | Spell it as it sounds | 2.65 | 41.17 | 0.44 | 0.01 | 0.05* | 0.5 | 0.44 | 0.02-0.11 |
|  | Nonword repetition | 1.19 | 41.06 | 0.2 | 0.24 | 0.49 | 0.5 | 0.47 | -0.02-0.08 |
|  | Digit span | 0.71 | 41.09 | 0.12 | 0.48 | 0.71 | 0.5 | 0.48 | -0.03-0.07 |
|  | Letter-number sequencing | 2.08 | 41.32 | 0.33 | 0.04 | 0.17 | 0.5 | 0.45 | 0-0.1 |
| Eating Disorder | Reading composite | 0.13 | 16.54 | 0.03 | 0.9 | 0.96 | 0.5 | 0.5 | -0.06-0.07 |
|  | Reading regular | -0.16 | 16.28 | 0.5 | 0.88 | 0.95 | 0.5 | 0.51 | -0.1-0.08 |
|  | Reading irregular | -0.29 | 16.43 | 0.07 | 0.78 | 0.90 | 0.5 | 0.51 | -0.08-0.06 |
|  | Reading nonwords | -0.07 | 16.48 | 0.02 | 0.94 | 0.98 | 0.5 | 0.5 | -0.07-0.07 |
|  | Spelling regular | 1.27 | 17.19 | 0.18 | 0.22 | 0.47 | 0.5 | 0.47 | -0.02-0.07 |
|  | Spelling irregular | -0.56 | 16.49 | 0.12 | 0.58 | 0.76 | 0.5 | 0.52 | -0.09-0.05 |
|  | Spell it as it sounds | 1.07 | 16.35 | 0.28 | 0.3 | 0.53 | 0.5 | 0.46 | -0.04-0.12 |
|  | Nonword repetition | 0.85 | 16.66 | 0.16 | 0.41 | 0.64 | 0.5 | 0.48 | -0.04-0.08 |
|  | Digit span | 0.69 | 15.41 | 0.16 | 0.5 | 0.69 | 0.5 | 0.48 | -0.05-0.1 |
|  | Letter-number sequencing | 2.4 | 15.76 | 0.41 | 0.03 | 0.12 | 0.5 | 0.44 | 0.01-0.11 |
| Personality Disorder | Reading composite | 0.77 | 4.01 | 0.55 | 0.48 | 0.72 | 0.5 | 0.42 | -0.21-0.37 |
|  | Reading regular | 0.09 | 4.01 | 0.08 | 0.93 | 0.98 | 0.5 | 0.49 | -0.36-0.39 |
|  | Reading irregular | 0.74 | 4.01 | 0.49 | 0.5 | 0.69 | 0.5 | 0.43 | -0.2-0.34 |
|  | Reading nonwords | 1 | 4.02 | 0.58 | 0.37 | 0.60 | 0.5 | 0.42 | -0.15-0.32 |
|  | Spelling regular | 1.38 | 4.02 | 0.73 | 0.24 | 0.50 | 0.5 | 0.4 | -0.11-0.32 |
|  | Spelling irregular | 1.57 | 4.03 | 0.68 | 0.19 | 0.46 | 0.5 | 0.4 | -0.08-0.27 |
|  | Spell it as it sounds | 3.77 | 4.02 | 1.87 | 0.02 | 0.08 | 0.5 | 0.23 | 0.07-0.48 |
|  | Nonword repetition | -0.29 | 4.02 | 0.15 | 0.79 | 0.89 | 0.5 | 0.52 | -0.23-0.19 |
|  | Digit span | 1.19 | 4.04 | 0.47 | 0.3 | 0.53 | 0.5 | 0.43 | -0.09-0.23 |
|  | Letter-number sequencing | -0.43 | 4.07 | 0.12 | 0.69 | 0.84 | 0.5 | 0.52 | -0.13-0.1 |
| Bipolar Disorder | Reading composite | 0.64 | 3.01 | 0.41 | 0.56 | 0.74 | 0.5 | 0.44 | -0.23-0.35 |
|  | Reading regular | 0.29 | 3.02 | 0.16 | 0.79 | 0.89 | 0.5 | 0.48 | -0.23-0.27 |
|  | Reading irregular | 0.79 | 3.01 | 0.57 | 0.49 | 0.71 | 0.5 | 0.42 | -0.25-0.42 |
|  | Reading nonwords | -0.59 | 3.01 | 0.35 | 0.6 | 0.76 | 0.5 | 0.55 | -0.33-0.22 |
|  | Spelling regular | 0.22 | 3.01 | 0.18 | 0.84 | 0.92 | 0.5 | 0.48 | -0.35-0.4 |
|  | Spelling irregular | 2.32 | 3.04 | 0.81 | 0.1 | 0.31 | 0.5 | 0.38 | -0.04-0.28 |
|  | Spell it as it sounds | 1.24 | 3.03 | 0.45 | 0.3 | 0.53 | 0.5 | 0.43 | -0.1-0.24 |
|  | Nonword repetition | -0.3 | 3.03 | 0.13 | 0.78 | 0.90 | 0.5 | 0.52 | -0.21-0.17 |
|  | Digit span | 1.39 | 3.01 | 0.81 | 0.26 | 0.49 | 0.5 | 0.38 | -0.15-0.39 |
|  | Letter-number sequencing | 2.08 | 3.04 | 0.68 | 0.13 | 0.36 | 0.5 | 0.4 | -0.05-0.25 |

**Supplementary Table 7:** Fishers exact test for enrichment of having at least one child diagnosed with a reading or language impairment for participants with a reading or language diagnosis compared to controls.

| **Parental diagnosis** | **Child diagnosis** | **p-value** | **OR** | **95% CI** |
| --- | --- | --- | --- | --- |
| Reading/ writing difficulties | Reading impairment | 1.23x10^-6^ | 3.31 | 2.03-5.34 |
|  | Language impairment | 0.08 | 2.03 | 0.8-4.51 |
| Reading difficulties | Reading impairment | 1.19x10^-5^ | 3.54 | 1.98-6.2 |
|  | Language impairment | 0.53 | 1.4 | 0.36-4.0 |
| Dyslexia | Reading impairment | 0.03 | 2.57 | 0.94-7.2 |
|  | Language impairment | 0.07 | 3.36 | 0.62-11.9 |
| Language impairment | Reading impairment | 6.32x10^-4^ | 3.66 | 1.67-7.73 |
|  | Language impairment | 0.07 | 2.83 | 0.7-8.44 |

**Supplementary Table 8:** Test for association between self-report of reading difficulties or language impairment in at least one biological child and composite reading ability using Welch's two sample t-test, *p ≤ 0.05. *Note*: quantitative scores were scaled to a mean of 0.5 for all samples (cases and controls).

| ***Child Diagnosis*** | ***t*** | ***df*** | ***Cohens d*** | ***P Unadjusted*** | ***control mean*** | ***case mean*** | ***95% CI*** |
| --- | --- | --- | --- | --- | --- | --- | --- |
| Reading impairment | 3.24 | 199.67 | 0.29 | 1.42x10^-03^ * | 0.507 | 0.465 | 0.063-0.067 |
| Language Impairment | -0.66 | 45.28 | 0.11 | 0.51 | 0.501 | 0.516 | -0.061-0.031 |

**Supplementary Table 9:** One way ANOVA tests (not assuming equal variance) for interviewer effects on normalised residual scores (df – degrees of freedom). Tukeys test of mean differences pairwise between interviewers, and p-value adjusted for multiple testing. *p ≤ 0.05, **p ≤ 0.01, ***p≤ 0.001.

| **Reading regular words** | F | df | | p-value |
| --- | --- | --- | --- | --- |
| Between interviewers | 52.33 | 3 | | <2.2x10^-16^ *** |
|  |  |  | |  |
| Pairwise interviewer comparison |  | Difference between means (95% CI) | | Adjusted p-value |
| Interviewer A | Interviewer B | -0.1 (±0.03) | | <1.0x10^-7^ *** |
| Interviewer A | Interviewer C | -0.03 (±0.03) | | 0.01 ** |
| Interviewer A | Interviewer D | -0.01 (±0.03) | | 0.85 |
| Interviewer B | Interviewer C | 0.07 (±0.03) | | <1.0x10^-7^ *** |
| Interviewer B | Interviewer D | 0.09 (±0.03) | | <1.0x10^-7^ *** |
| Interviewer C | Interviewer D | 0.03 (±0.03) | | 0.13 |
|  |  |  | |  |
| **Reading irregular words** | F | df | | p-value |
| Between interviewers | 34.4 | 3 | | <2.2x10^-16^ *** |
|  |  |  | |  |
| Pairwise interviewer comparison |  | | Difference between means (95% CI) | Adjusted p-value |
| Interviewer A | Interviewer B | 0.01 (±0.03) | | 0.72 |
| Interviewer A | Interviewer C | 0.05 (±0.03) | | 8.4x10^-5^ *** |
| Interviewer A | Interviewer D | 0.1 (±0.03) | | <1.0x10^-7^ *** |
| Interviewer B | Interviewer C | 0.04 (±0.03) | | 1.4x10^-3^ ** |
| Interviewer B | Interviewer D | 0.08 (±0.03) | | <1.0x10^-7^ *** |
| Interviewer C | Interviewer D | 0.05 (±0.03) | | 3x10^-3^ ** |
|  |  |  | |  |
| **Reading nonwords** | F | df | | p-value |
| Between interviewers | 37.78 | 3 | | <2.2x10^-16^ *** |
|  |  |  | |  |
| Pairwise interviewer comparison |  | Difference between means (95% CI) | | Adjusted p-value |
| Interviewer A | Interviewer B | 0.01 (±0.03) | | 0.43 |
| Interviewer A | Interviewer C | 0.01 (±0.03) | | 0.73 |
| Interviewer A | Interviewer D | 0.1 (±0.03) | | <1.0x10^-7^ *** |
| Interviewer B | Interviewer C | -2.73x10^-3^ (±0.03) | | 0.99 |
| Interviewer B | Interviewer D | 0.09 (±0.03) | | <1.0x10^-7^ *** |
| Interviewer C | Interviewer D | 0.09 (±0.03) | | <1.0x10^-7^ *** |
|  |  |  | |  |
| **Spelling regular words** | F | df | | p-value |
| Between interviewers | 27.8 | 3 | | <2.2x10^-16^ *** |
|  |  |  | |  |
| Pairwise interviewer comparison |  | Difference between means (95% CI) | | Adjusted p-value |
| Interviewer A | Interviewer B | 0.08 (±0.03) | | <1.0x10^-7^ *** |
| Interviewer A | Interviewer C | 0.05 (±0.03) | | 3.33x10^-4^ *** |
| Interviewer A | Interviewer D | 0.09 (±0.03) | | <1.0x10^-7^ *** |
| Interviewer B | Interviewer C | -0.03 (±0.03) | | 0.03 * |
| Interviewer B | Interviewer D | 0.01 (±0.03) | | 0.74 |
| Interviewer C | Interviewer D | 0.04 (±0.03) | | 4.17x10^-3^ ** |
|  |  |  | |  |
| **Spelling irregular words** | F | df | | p-value |
| Between interviewers | 5.0 | 3 | | 1.9x10^-3^ ** |
|  |  |  | |  |
| Pairwise interviewer comparison |  | Difference between means (95% CI) | | Adjusted p-value |
| Interviewer A | Interviewer B | 3.79x10^-2^ (±0.03) | | 9.49x10^-4^ *** |
| Interviewer A | Interviewer C | 3.05x10^-2^ (±0.03) | | 0.04 * |
| Interviewer A | Interviewer D | 3.05x10^-2^ (±0.03) | | 0.03 * |
| Interviewer B | Interviewer C | -7.4x10^-3^ (±0.03) | | 0.91 |
| Interviewer B | Interviewer D | -7.4x10^-3^ (±0.03) | | 0.9 |
| Interviewer C | Interviewer D | -8.24x10^-5^ (±0.03) | | 1 |
|  |  |  | |  |
| **Spell it as it sounds** | F | df | | p-value |
| Between interviewers | 1.66 | 3 | | 0.17 |
|  |  |  | |  |
| **Nonword repetition** | F | df | | p-value |
| Between interviewers | 337.13 | 3 | | <2.2x10^-16^ *** |
|  |  |  | |  |
| Pairwise interviewer comparison |  | Difference between means (95% CI) | | Adjusted p-value |
| Interviewer A | Interviewer B | -0.06 (±0.02) | | <1.0x10^-7^ *** |
| Interviewer A | Interviewer C | 0.1 (±0.02) | | <1.0x10^-7^ *** |
| Interviewer A | Interviewer D | 0.19 (±0.02) | | <1.0x10^-7^ *** |
| Interviewer B | Interviewer C | 0.16 (±0.02) | | <1.0x10^-7^ *** |
| Interviewer B | Interviewer D | 0.24 (±0.02) | | <1.0x10^-7^ *** |
| Interviewer C | Interviewer D | 0.08 (±0.02) | | <1.0x10^-7^ *** |
|  |  |  | |  |
| **Digit Span** | F | df | | p-value |
| Between interviewers | 2.5 | 3 | | 0.06 |
|  |  |  | |  |
| **Letter-number sequencing** | F | df | | p-value |
| Between interviewers | 90.52 | 3 | | <2.2x10^-16^ *** |
|  |  |  | |  |
| Pairwise interviewer comparison |  | Difference between means (95% CI) | | Adjusted p-value |
| Interviewer A | Interviewer B | -0.13 (±0.02) | | <1.0x10^-7^ *** |
| Interviewer A | Interviewer C | 4.33x10^-3^ (±0.03) | | 0.98 |
| Interviewer A | Interviewer D | -0.03 (±0.03) | | 0.01 ** |
| Interviewer B | Interviewer C | 0.13 (±0.03) | | <1.0x10^-7^ *** |
| Interviewer B | Interviewer D | 0.09 (±0.03) | | <1.0x10^-7^ *** |
| Interviewer C | Interviewer D | -0.04 (±0.03) | | 6.99x10^-3^ ** |

**Supplementary Table 10:** Pearson correlations between raw and ANOVA adjusted measures corrected for interviewer.

| **Measure** | **Correlation (Pearson r)** | **P value** |
| --- | --- | --- |
| Regular word reading | 0.97 | <2.2x10^-16^ |
| Irregular word reading | 0.97 | <2.2x10^-16^ |
| Nonword reading | 0.98 | <2.2x10^-16^ |
| Regular spelling | 0.97 | <2.2x10^-16^ |
| Irregular spelling | 0.99 | <2.2x10^-16^ |
| Spell it as it sounds | 1 | <2.2x10^-16^ |
| Nonword repetition | 0.82 | <2.2x10^-16^ |
| Digit span | 1 | <2.2x10^-16^ |
| Letter-number sequencing | 0.92 | <2.2x10^-16^ |
