# Supplementary material for: Measuring reading and language skill in generation Scotland: Scottish Family Health Study
Source: BMJ Public Health. 2026 May 21;4(2):e004427. doi: 10.1136/bmjph-2025-004427 (PMC13202090; doi:10.1136/bmjph-2025-004427)
Supplement: online supplemental file 3 [file bmjph-4-2-s003.docx]

**SUPPLEMENTARY NOTES**

***Generation Scotland: Scottish Family Cohort Study (GS:SFHS)***

Generation Scotland: Scottish Family Cohort Study (GS:SFHS) is an extended pedigree study of 24,084 adults from 5501 families that started in 2006 with baseline collections of cognitive and health data, and laboratory sampling and molecular assays of various biomarkers (including genomics, methylomics, proteomics and metabolomics) [1]. Since then, subsamples of participants have participated in bespoke studies often focusing on specific topics of interest. These include STRADL [2], a study of depression that includes measurement of brain magnetic resonance imaging; DOLORisk, a study of neuropathic pain [3], COVIDLife, a study of health and wellbeing during the COVID pandemic [4]; and partnerships with other studies focussed on the prevention of Alzheimer disease [5] and healthy ageing in Scotland [6]. Furthermore, the GS:SFHS cohort benefits from existing and future planned linkages to NHS datasets and administrative data, for instance, education records.

***Online Self-Report Questionnaire***

Thank you for agreeing to be involved in the GS Reading Study

We greatly value your contribution to our research. This is a confidential survey consisting of a few brief questions related to reading and language. Please answer all questions as freely and honestly as possible and complete the questionnaire on your own.

1. Have you ever had any difficulties with reading or writing? If yes, were these:
   1. Self-evaluated
   2. Noticed by others (e.g. teachers, parents)
   3. Clinically diagnosed
      1. If clinically diagnosed, what was the diagnosis?
2. Have you ever had any other language impairment? If yes, were these:
   1. Self-evaluated
   2. Noticed by others (e.g. teachers, parents)
   3. Clinically diagnosed
      1. If clinically diagnosed, what was the diagnosis?
3. Do you stammer/ stutter?
   1. No
   2. Not now, but in the past
   3. Yes
4. How often do you read a book?
   1. Less than once a year/ never
   2. Several times a year
   3. Several times a month
   4. Several times a week
   5. Every day or about every day
5. How often did you read a book as a child?
   1. Less than once a year/ never
   2. Several times a year
   3. Several times a month
   4. Several times a week
   5. Every day or about every day
6. Have you ever been diagnosed with any of the following (yes/ no)?
   1. Developmental delay
   2. Dyspraxia
   3. Autism Spectrum Condition (including Asperger Syndrome)
   4. Hyperactivity/ Attention Deficit Disorder (ADHD, ADD)
   5. Tourette’s Syndrome
   6. Eating Disorder
   7. Obsessive Compulsive Disorder
   8. Depression
   9. Bipolar Disorder
   10. Social Anxiety Disorder
   11. Personality Disorder
7. Do you have any biological children (yes/ no)?
8. If yes: To help us with future follow-up studies, we would like to ask two questions about your biological children. Would you like to continue (yes/ no)?
9. Have any of your biological children been diagnosed with a reading impairment (yes/ no)?
   1. If yes, what was the diagnosis?
10. Have any of your biological children been diagnosed with a reading impairment (yes/ no)?
    1. If yes, what was the diagnosis?

Thank you for your help!

One of our interviewers will be in touch with you over the next few days to arrange a telephone interview.

***Quantitative Test Battery***

Interviews were conducted by a trained interviewer over the telephone, following the method described in Doust, Gordon [7].

Participants were requested to be in a quiet environment with no distractions, and to ensure a good quality telephone signal. Difficulties with telephone line quality, background noise or hearing difficulties were noted by the interviewers.

Reading: Single word reading ability was assessed using the Castles and Coltheart test 2 adults (CC2A) [8]. The CC2A presents a regular, irregular, and non-word group of three words in a randomised order, with each group increasing in difficulty. Regular, irregular and non-word reading scored out of 55, and a total reading score of 165. Words were presented online by participants clicking on a link sent to their email at the time of interview; correct or incorrect response was recorded in Testable by the interviewer with a stopping rule of 5 consecutive incorrect responses. Participants were requested to use a computer or tablet, rather than a mobile phone to ensure that the words were clearly visible.

Spelling: Spelling ability was examined using the Components of Reading Examination spelling test [9]. The test presented 14 regular and 14 irregular words increasing in difficulty. A “spell it as it sounds” regularised spelling measure required the participant to spell 14 words phonetically [9]. Words were read out loud by the interviewer, and the response as spelled by the participant was recorded in Qualtrics.

Nonword Repetition: Nonword repetition is considered a marker of phonological short-term memory and has been linked to variation in oral language ability [10]. The non-word repetition task used here, known as CNRep [11], presents 40 nonsense words consisting of two to five syllables of speech-sounds in novel combinations in increasing difficulty. Words were read out loud by the interviewer, and then whether the response was correct or incorrect was recorded.

Auditory short-term and working memory: Digit span forwards and letter-number sequencing tests from the WAIS-III [12] were used to assess respective verbal auditory short-term and working memory. Items were read aloud by the interviewer, and participant responses were recorded as correct or incorrect in Qualtrics.

***Participant exclusion reasons***

Prior to commencing the study, 61 participants reported as having hearing difficulties that may impact their ability to hear instructions over the telephone. Seven were excluded prior to commencing the study as their ability to hear instructions over the phone was too severely impacted. Participants were instructed to wear their hearing aid if required, and to ensure they could hear appropriately.

Notes taken by the tester during the interview as well as the self-report questionnaire were examined for reading difficulties, neurodevelopmental or psychiatric conditions, or other factors which may explain a low-test score. Individuals were excluded from the dataset if they reported a medical condition that severely impacted their ability to participate or to hear the interviewer (N = 4).

Reasons for exclusion from study post-testing (4 participants):

- Neurological conditions affecting concentration and memory (N = 1)
- Functional neurological disorder due to spinal surgery (N = 1)
- Reported severe hearing and memory difficulties (N = 1)
- Reported severe hearing difficulties and could not hear tester (N = 1)

Participants were excluded from individual tests if they reported problems that specifically interfered with that test e.g., did the reading test on the phone and could not see words, poor phone line prevented them from hearing the words, or they misunderstood the instructions of the test instead noting down components of the digit and letter span items rather than memorising them.

Excluded from reading tests (1 participant) because:

- Participant did the reading test on a mobile phone, and numbers were over the words so she could not see them

No participants were excluded from spelling tests

Excluded from “spell it as it sounds” (2 participants) because:

- Both had very poor-quality phone lines which severely affected ability to hear words

Excluded from Nonword repetition tests (5 participants) because:

- Five had very poor phone lines that affected their ability to hear words

Excluded from WAIS-III tests (8 participants) because:

- Five participants misunderstood the instructions and wrote down numbers and letters
- Three had very bad phone line affected ability to hear letters and digits

***Extended Statistical Methods***

Multiple regression: We applied a linear model defined as *Adjusted outcome ~ sex + age + age^2^* to the quantitative variables. This regression analysis assumes the observations are independent, outcome variable is normally distributed, homoscedasticity and the absence of multicollinearity.

Phenotype correlations: Correlations were examined using *mixedCor* in the *psych* R package [13] which determines which pairs of variables are to be correlated using Pearson, polyserial or polychoric methods based on variable type. All complete pairs were used for analysis. P values and confidence intervals were bootstrapped using a script adapted from the *cor.ci* function (*psych*) [13] for continuous and discrete variables, and false discovery rate (FDR) corrected p-values are reported using the Benjami-Hochberg method [14].

Composite measures of reading ability: To obtain an overall view of reading and reading-related aspects, a composite score of reading ability was generated using the unscaled residuals from regular, irregular and nonword reading tests, and regular and irregular spelling tests all adjusted for sex, age and age^2^. Principal component analysis was performed without rotation, using *principal* (*psych*) [13] using only individuals with complete datapoints (N = 1466). The appropriate number of components was determined by scree plot (*psych*) [13]. Composite scores were extracted using the loadings from the first-principal component, then inverse normal transformed and rescaled from 0-1.

Cohort validation: Welch's two sample t-test (implemented using the *t.test* function in R) [15] was used to compare the means of the normalised quantitative measures between groups defined by self-reported neurodevelopmental and psychiatric conditions. This test was selected as it does not assume equal variance between groups. The hypothesis for Welch’s two sample t-test [15] are:

- Null hypothesis (**H_o_**) indicating no difference in group means: μ_1_ = μ_2_
- Alternative hypothesis (**H_1_**) indicating a difference in group means: μ_1_ ≠ μ_2_

The test statistic (**t**) was calculated as follows, where *X* is sample mean, *s* is sample variance (square of standard deviation), *n* is sample size:

$$t= \frac{\bar{X}_{1}-\bar{X}_{2}}{\sqrt{\frac{s_{1}^{2}}{n_{1}}}+ \frac{s_{2}^{2}}{n_{2}}}$$

Degrees of freedom (**df**) were approximated using the Welch-Satterthwaite equation as follows:

$$df= \frac{\left( \frac{s_{1}^{2}}{n_{1}}+ \frac{s_{2}^{2}}{n_{2}} \right)^{2}}{\frac{\left( \frac{s_{1}^{2}}{n_{1}} \right)^{2}}{n_{1}-1}+ \frac{\left( \frac{s_{2}^{2}}{n_{2}} \right)^{2}}{n_{2}-1}}$$

Cohens d (***d***) [16] were calculated in the R package *lsr* [17] using the equation below, where *X* is sample mean, *n* is sample size *s_p_* indicates the pooled standard deviation:

$$d= \frac{\bar{X}_{1}-\bar{X}_{2}}{s_{p}}$$

To account for multiple testing, p-values were adjusted using Benjami-Hochberg method FDR correction [14]. The corrected p-values are reported. Associations were considered statistically significant at an FDR-adjusted threshold of 0.05.

Reading and language difficulties in biological children: Associations between self-reported reading or language difficulties in study participants and the presence of difficulties in their biological children were evaluated using Fisher's Exact Test for count data (implemented by *fisher.test* in R) [18]. Fisher’s Exact Test was used to assess associations between two categorical variables in 2×2 contingency tables. This test was selected to account for small sample sizes and low expected counts. P-values were reported, and statistical significance was assessed at a threshold of 0.05.

- Null hypothesis (**H_o_**): The two categorical variables are independent (no association).
- Alternative hypothesis (**H_1_**): The two categorical variables are associated (not independent).
- Reject **H_o_** if p < 0.05, or do not reject **H_o_** if p ≥ 0.05

Association between self-report of reading difficulties or language impairment in at least one biological child and composite reading ability was performed using Welch's two sample t-test as previously described.

Interviewer effects: Interviewer effects on quantitative measures were examined using one-way ANOVA (*oneway.test*), followed by Tukey's Honestly Significant Difference (HSD) test for multiple comparisons (*TukeyHSD*). Pearson correlations between raw and interviewer adjusted measures were performed using *cor.test* (*psych*) and visualised as previously described.

***Frequency of Book Reading***

Frequent book reading as a child showed a moderate, significant correlation, with frequency of book reading as an adult (0.53, p = 0). Higher scores in reading, spelling and spell it as it sounds were significantly correlated with more frequent book reading as a child (≥0.2, p ≤8.33x10^-6^) and as an adult (≥0.14, p ≤3.37x10^-10^). The strongest correlations were observed between higher scores on irregular word reading and more frequent book reading as a child (0.39, p = 0) and as an adult (0.28, p = 0). Nonword repetition was significantly correlated with higher reading frequency as an adult (0.1, p = 1.55x10^-3^) and as a child (0.12, p = 2.22x10^-5^). Digit span was significantly correlated with reading frequency as an adult (0.1, p = 2.89x10^-3^) and a child (0.12, p = 3.91x10^-5^), whereas letter-number sequencing was only significantly correlated as a child (0.11, p = 2.3x10^-4^) [**SUPP TABLES 3 and 4**].

Significant correlations were observed between individuals with self-reported reading and/or writing difficulties and frequency of book reading as a child (-0.3, p = 1.03x10^-7^) and an adult (-0.28, p = 1.23x10^-7^). Similarly, reading difficulties and reading less frequently as a child (-0.38, p = 1.13x10^-8^) and at the time of testing (-0.32, p = 4.5x10^-7^) was significantly correlated, as was dyslexia (child: -0.26, p = 0.01; adult: -0.29, p = 2.78x10^-3^). Stuttering was correlated with lower frequency of book reading in both childhood (-0.23, p = 4.05x10^-3^) and adulthood (-0.2, p = 0.02), as was social anxiety disorder (child: -0.16, p = 0.05; adult: -0.2, p = 0.02).

Self-reports of dyspraxia (child: -0.33; adult -0.13) and autism (child -0.55; adult -0.4) showed negative correlations with more frequent reading, albeit nonsignificant. Developmental delay showed a negative (nonsignificant) correlation with reading frequency as a child (-0.37) but not as an adult (0.03). Self-reports of ADHD showed a positive correlation with reading frequency as a child (0.17) and a negative correlation as an adult (-0.15), both nonsignificant. OCD showed a negative nonsignificant correlation with reading frequency as a child (-0.2), but not as an adult (0.03), and language impairments showed no correlation either in childhood (-0.07) or as an adult (-0.06).

***Interviewer differences***

To test for any interviewer biases, we plotted the normalised scores for nine quantitative variables separated by interviewer [**SUPP TABLE 9**]. One way ANOVA indicated there were statistically significant differences between the mean scores of at least two of the interviewers in all quantitative measures except for Spell it as it sounds (F = 1.66, p = 0.17) and Digit Span (F = 2.5, p = 0.06). Tukey’s pairwise test of mean differences showed that no individual interviewer was consistently different across reading, spelling or all tests [**SUPP TABLE 9**]. For example, the mean difference between Interviewer B and the other three was ≥0.07 (p <1.0x10^-7^) on regular word reading (i.e. 7% of the normalised score), however on irregular word reading Interviewers C and D were significantly different by ≥0.04 (p <1.4x10^-3^) and ≥0.01 (p <1.0x10^-7^), consecutively, while also differing 0.05 (p = 3x10^-3^) from each other. The highest degree of interviewer bias was detected on nonword repetition, where all four interviewers differed significantly from each other with mean differences of between 0.06 to 0.24 (p <1.0x10^-7^).

To visually inspect these potential interviewer biases, we corrected the raw scores for interviewer using ANOVA and plotted correlation matrices of neurodevelopmentally relevant binary variables [**SUPP FIGURE 9**]. Pearson correlations between raw and interviewer adjusted measures were ≥0.9 (p <2.2x10^-16^) except for nonword repetition which was at 0.82 (p <2.2x10^-16^), indicating there is minimal interviewer biases across the dataset [**SUPP TABLE 10**].

***References***

1. Milbourn H, McCartney D, Richmond A, Campbell A, Flaig R, Robertson S, et al. Generation Scotland: an update on Scotland's longitudinal family health study. BMJ Open. 2024;14(6):e084719. doi:10.1136/bmjopen-2024-084719.

2. Habota T, Sandu AL, Waiter GD, McNeil CJ, Steele JD, Macfarlane JA, et al. Cohort profile for the STratifying Resilience and Depression Longitudinally (STRADL) study: A depression-focused investigation of Generation Scotland, using detailed clinical, cognitive, and neuroimaging assessments. Wellcome Open Res. 2019;4:185. doi:10.12688/wellcomeopenres.15538.2.

3. Hebert HL, Veluchamy A, Baskozos G, Fardo F, Van Ryckeghem DML, Pascal MMV, et al. Cohort profile: DOLORisk Dundee: a longitudinal study of chronic neuropathic pain. BMJ Open. 2021;11(5):e042887. doi:10.1136/bmjopen-2020-042887.

4. Fawns-Ritchie C, Altschul DM, Campbell A, Huggins C, Nangle C, Dawson R, et al. CovidLife: a resource to understand mental health, well-being and behaviour during the COVID-19 pandemic in the UK. Wellcome Open Res. 2021;6:176. doi:10.12688/wellcomeopenres.16987.1.

5. Solomon A, Kivipelto M, Molinuevo JL, Tom B, Ritchie CW, Consortium E. European Prevention of Alzheimer's Dementia Longitudinal Cohort Study (EPAD LCS): study protocol. BMJ Open. 2019;8(12):e021017. doi:10.1136/bmjopen-2017-021017.

6. Douglas E, Rutherford A, Bell D. Pilot study protocol to inform a future longitudinal study of ageing using linked administrative data: Healthy AGeing in Scotland (HAGIS). BMJ Open. 2018;8(1):e018802. doi:10.1136/bmjopen-2017-018802.

7. Doust C, Gordon SD, Garden N, Fisher SE, Martin NG, Bates TC, et al. The Association of Dyslexia and Developmental Speech and Language Disorder Candidate Genes with Reading and Language Abilities in Adults. Twin Res Hum Genet. 2020;23(1):23-32. doi:10.1017/thg.2020.7.

8. Castles A, Coltheart M, Larsen L, Jones P, Saunders S, McArthur G. Assessing the basic components of reading: A revision of the Castles and Coltheart test with new norms. Australian Journal of Learning Difficulties. 2009;14(1):67-88. doi:10.1080/19404150902783435.

9. Bates TC, Castles A, Coltheart M, Gillespie NA, Wright MJ, Martin NG. Behaviour genetic analyses of reading and spelling: A component processes approach. Australian Journal of Psychology. 2004;56(2):115-26. doi:10.1080/00049530410001734847.

10. Gathercole SE, Baddeley AD. Phonological memory deficits in language disordered children: Is there a causal connection? Journal of Memory and Language. 1990;29(3):336-60. doi:10.1016/0749-596X(90)90004-J.

11. Gathercole SE, Willis CS, Baddeley AD, Emslie H. The children's test of nonword repetition: A test of phonological working memory. Memory. 1994;2(2):103-27. doi:10.1080/09658219408258940.

12. Wechsler D. Wechsler Adult Intelligence Scale--Third Edition (WAIS-III). APA PsycTests. 1997. doi:10.1037/t49755-000.

13. Revelle W. psych: Procedures for Psychological, Psychometric, and Personality Research version 2.5.3. CRAN: Contributed Packages. 2025. doi:10.32614/CRAN.package.psych.

14. Benjamini Y, Hochberg Y. Controlling the False Discovery Rate: A Practical and Powerful Approach to Multiple Testing. Journal of the Royal Statistical Society: Series B (Methodological). 1995;57(1):289-300. doi:10.1111/j.2517-6161.1995.tb02031.x.

15. Welch BL. The generalisation of student's problems when several different population variances are involved. Biometrika. 1947;34(1-2):28-35. doi:10.1093/biomet/34.1-2.28.

16. Cohen J. A power primer. Psychol Bull. 1992;112(1):155-9. doi:10.1037//0033-2909.112.1.155.

17. Navarro D. Learning statistics with R: A tutorial for psychology students and other beginners Version 0.6. CRAN: Contributed Packages. 2015. doi:<http://ua.edu.au/ccs/teaching/lsr>.

18. Upton GJG. Fisher's Exact Test. Journal of the Royal Statistical Society Series A (Statistics in Society). 1992;155(3):395-402. doi:10.2307/2982890.
